# Effect of bariatric surgery in the body burden of persistent and non-persistent pollutants: longitudinal study in a cohort of morbidly obese patients

**DOI:** 10.3389/fendo.2024.1412261

**Published:** 2024-07-22

**Authors:** B. Vanessa Díaz-González, Álvaro Ramos-Luzardo, Luis Alberto Henríquez-Hernández, Lluis Serra-Majem, Inmaculada Bautista-Castaño, Andrea Acosta-Dacal, Octavio P. Luzardo, Elisabeth Hernández-García, Judith Cornejo-Torre, Juan Ramón Hernández-Hernández, Pilar Fernández-Valerón

**Affiliations:** ^1^ Triana Primary Health Care Center, Servicio Canario de la Salud, Las Palmas de Gran Canaria, Spain; ^2^ Research Institute of Biomedical and Health Sciences (IUIBS), Universidad de Las Palmas de Gran Canaria, Las Palmas de Gran Canaria, Spain; ^3^ Department of Biochemistry and Molecular Biology, Physiology, Genetics, and Immunology, Universidad de Las Palmas de Gran Canaria, Las Palmas de Gran Canaria, Spain; ^4^ Toxicology Unit, Clinical Sciences Department, Universidad de Las Palmas de Gran Canaria, Las Palmas de Gran Canaria, Spain; ^5^ Centro de Investigación Biomédica en Red Fisiopatología de la Obesidad y la Nutrición (CIBEROBN), Instituto de Salud Carlos III, Madrid, Spain; ^6^ Centro Hospitalario Universitario Insular Materno Infantil (CHUIMI), Servicio Canario de la Salud, Las Palmas de Gran Canaria, Spain

**Keywords:** obesity, bariatric surgery, persistent organic pollutants, weight loss, obesogens

## Abstract

**Introduction:**

Obesity is a pathological state that involves the dysregulation of different metabolic pathways and adipose tissue cells, constituting a risk factor for the development of other diseases. Bariatric surgery is the most effective treatment. The study of the behavior of pollutants in situations of extreme weight loss can provide biomonitoring information and tools to manage diseases of environmental etiology.

**Aim:**

To determine the prevalence of serum persistent and non-persistent pollutants in obese patients subjected to bariatric surgery and analyze the impact of sociodemographic variables on these changes.

**Methods:**

GC-MS/MS and UHPLC-MS/MS were utilized to determine the detection rates and concentrations of 353 compounds, including persistent organic pollutants (POPs), pesticides, pharmaceuticals, and rodenticide, in serum samples of 59 obese patients before and after undergoing bariatric surgery.

**Results:**

Detection rates of p,p’-DDE, HCB, β-HCH, naphthalene, phenanthrene and PCB congeners 138, 153 and 180 significantly increased due to surgery-induced weight loss. Serum levels of p,p’-DDE, PCB-138, PCB-153 and PCB-180 also increased after surgery. Correlations between naphthalene levels, weight loss, variation of total lipids and time after surgery were found. Additionally, correlations were observed between concentrations of PCB-138 and weight loss, and between phenanthrene levels and reduction of total lipids. No statistically significant differences were observed for other groups of contaminants, pharmaceuticals and other chemicals included in the quantification methods.

**Conclusions:**

Increment of POPs was observed after bariatric surgery. Serum concentrations of POPs after surgery were influenced by adiposity-related variables. Although biomonitoring studies show a decreasing tendency of exposure, rapid weight loss leads to an increase of circulating POPs. Further research on the interplay between adipose tissue, POPs and peripheral organs is required.

## 1 Introduction

During the last three decades, the prevalence of obesity has doubled worldwide (1, 2). Due to this fact, it is now considered the pandemic of the modern era, which carries a huge socioeconomic impact (1, 3). According to the European Regional Report on Obesity, published by the WHO in May 2022, 60% of the European adult population are affected either by overweight or obesity (4). This chronic physical condition is characterized by an increased body mass index (BMI, expressed as kg/m^2^) that allows for its classification in different somatotypes, namely overweight (25 ≤ BMI <30), class I obesity (30 ≤BMI <35), class II (35 ≤ BMI <40) and class III or severe obesity (BMI ≥ 40) (5). It is a multifactorial disease that originates as a result of the combination of intrinsic, physiological and behavioral causes together with extrinsic, lifestyle, dietary and exposure factors (3). Although this disease was primarily related to excessive fat accumulation in the adipose tissue, growing evidence points to whole-body low-grade chronic inflammation, dysregulation of the gut microbiota, alterations of the cell cycle in adipocytes and elevated levels of reactive oxygen species as important hallmarks of obesity (6, 7). Thus, rather than an imbalance of calories in and out, the underlying causes of obesity might involve the destabilization of energy homeostasis mechanisms themselves (3). Beyond the physical limitations that increased body mass involves, obesity has been associated with a set of comorbidities and complications, such as dyslipidemia, depression, cancer, type II diabetes, cardiovascular diseases and susceptibility to severe infections, i.e. SARS-CoV-2 (8–11).

Substantial progress has been made in the treatment of obesity over the last decades. This includes lifestyle interventions by restructuring eating and physical activity habits; pharmacological therapies targeting either digestive lipases or the central nervous system (CNS); administration of oral hydrogel capsules that induce the feeling of satiety; slightly invasive endoscopic bariatric therapies, such as intragastric balloons and endoscopic gastroplasty; and bariatric surgery, which shows the strongest evidence of sustained weight loss (12, 13). In spite of the promising results of pharmacotherapeutic strategies in clinical trials, bariatric surgery is still the most effective approach to achieve long-term body fat reduction, reducing the incidence of type 2 diabetes mellitus and obesity-associated cancer (12, 14). In general terms, bariatric surgery is indicated when there has been a poor response to other treatments, in patients with class 2 obesity with complications, or patients with class 3 obesity.

Recent advances in the research of obesity and the adipose tissue has reassessed it from being merely an storage tissue to a complex, metabolically active organ with different endocrine functions (15). Hence, the adipose tissue plays an important toxicological role with regard to xenobiotic lipophilic molecules. Firstly, it exerts a protective effect by sequestering hydrophobic chemicals from the bloodstream. Secondly, the adipose tissue is considered an internal source of low-concentration chronic exposure to stored xenochemicals, which may be released during lipolysis. Thirdly, adipocytes exhibit several receptors that can be either activated or inhibited by the interaction with these compounds, thereby constituting a key target for their activity (16) and reinforcing the obesogen theory (17). Interestingly, the association between adiposity and the presence of persistent organic pollutants (POPs) have been reported in previous studies, in which plasma levels of these contaminants were not only related to BMI and gender, but also appeared to increase in response to rapid weight loss (18), suggesting a transference between the adipose tissue and the circulatory system.

POPs are toxic organic molecules with some physicochemical properties that make them especially resistant to degradation and very susceptible to bioaccumulation and biomagnification (19). Therefore, their release into circulation could lead to undesired consequences. Even though most POPs are listed in the Stockholm Convention (20) and they are banned or under strict regulation in many countries, they are currently detected in the individuals due to their ubiquitous nature, their persistence in the environment and, in some cases, their transgenerational inheritance (19, 21). According to data, exposure to POPs is a contributing factor to different pathological conditions including obstetric defects, cardiovascular disease, metabolic syndrome, neurological and immunological disorders and cancer (8, 22–28). In the Spanish population, the levels of some POPs have decreased since their prohibition in the late 20^th^ century (29–31). However, exposure to these chemicals in the Canary Islands –a Spanish archipelago located near the west African coast– was proven to be higher than in the continental Europe (26). Despite the wide knowledge about the prevalence of these contaminants in this North Atlantic region, little is known about the situation in the obese non-elderly inhabitants. Moreover, the role of the bariatric surgery-induced weight loss in relation to these substances is unexplored.

This study aims to determine the serum levels of 353 compounds, including POPs, pesticides, pharmaceuticals, and rodenticides, in a cohort of obese patients from Gran Canaria Island (Spain), and stablish a comparison within samples from the same patients, collected one year after undergoing bariatric surgery. Additionally, the association between the resulting concentrations and sociodemographic factors were analyzed.

## 2 Materials and methods

### 2.1 Study population and sample collection

A total of 59 adult patients were included in this study, composed of 43 women and 16 men aged from 20 to 69 years old whose BMI ranged from 35 to 60 kg/m^2^. The patients underwent a bariatric surgery, preceded by a clinical evaluation, from March, 30^th^ to July, 7^th^, 2015, after which they followed a strict dietary control for one year and submitted to a second evaluation from March, 15^th^ to December, 23^rd^, 2016. Weight, height, and body composition were recorded using the Tanita BC-420MA III (Girod Medical, Madrid, Spain) before the intervention and during the follow-up visits. At the time of the initial visit and the final assessment, whole blood was collected in 4 mL tubes with EDTA and centrifuged at 3000g for 5 min. The serum was separated within a maximum of 2 h after collection. The obtained serum was kept at −20 °C until chemical analysis. We reported weight loss outcomes as total body weight loss (TBWL, expressed in kilograms), percentage of total body weight loss (%TBWL) and percentage of excess body weight loss (%EWL). Clinically relevant parameters, such as lipidemia, smoking habit, presence of comorbidities and depressive state, were measured both before and after the follow-up (**Table 1**).

**Table 1:** Characteristics of patients before surgery (n = 59).

	N	(%)
Gender		
Male	16	27.1
Female	43	72.9
Age (years)		
Mean ± SD	43.4 ± 13.1	
Median	43.0	
≤30	12	20.3
30 - 45	23	39.0
>45	24	40.7
Weight (kg)		
Mean ± SD	129.7 ± 24.2	
Median	127.5	
BMI (kg/m^2^)		
Mean ± SD	47.7 ± 6.5	
Median	48.3	
Obesity (grade II)	9	15.3
Obesity (grade III)	50	84.7
Smoking habit (yes)	10	16.9
Type of surgery		
Tube gastrectomy	38	64.4
Biliopancreatic diversion	8	13.6
Duodenal switch	12	20.3
Gastric bypass	1	1.7
Diabetes (yes)	23	39.9
Arterial hypertension (yes)	25	43.1
Total lipids (mg/mL)		
Mean ± SD	6.6 ± 0.9	
Median	6.5	

SD, standard deviation; BMI, body mass index.

The study was approved by the Clinical Research Ethics Committee of the CHUIMI (Complejo Hospitalario Universitario Insular Materno Infantil): ethical approval code 2024-093-728, on June 26^th^, 2014.

### 2.2 Sample preparation

Samples underwent a QuEChERS-based protocol for the extraction of analytes of interest (32), which have been modified and validated for the analysis of analytes in serum (33). Briefly, 250 μL of serum samples were introduced in 2 mL Eppendorf tubes and 10 μL of a mixture of procedural internal standards (P-ISs) were then added to reach a final concentration of 4 ng/μL of acenaphthene-d10, chlorpyrifos-d10, chrysene-d12, diazinon-d10, PCB-200, and phenanthrene-d10 for Gas Chromatography (GC); and atrazine-d5, carbendazim-d3, cyromazine-d4, diazinon-d10, linuron-d3 and pirimicarb-d6 for Liquid Chromatography (LC). After a 10-minute incubation at room temperature, 500 μL of acidified acetonitrile (1% formic acid (FA)) were added and properly mixed by vortexing. The serum-acetonitrile mixture underwent an ultrasonic bath (Selecta, Barcelona, Spain) for 20 minutes at room temperature, after which 150 mg of anhydrous magnesium sulfate and 37.5 mg of sodium acetate were added. Samples were vortexed and vigorously shaken for one minute and centrifuged at 4200 rpm and 4°C for 10 minutes in a centrifuge 5804/R with a rotor FA-45-48-11 (Eppendorf, Hamburg, Germany). The resulting supernatant was collected with a 1-mL syringe and introduced in an inserted chromatography amber vial using a polyester, HPLC-certified, 0.2μm Chromafil PET-20/15 MS syringe filter (Macherey-Nagel, Düren, Germany).

Applying this method, clean-up, evaporation and solvent change steps are avoided and 353 analytes can be detected and quantified through gas chromatography coupled to triple quadrupole mass spectrometry (GC-MS/MS) and liquid chromatography coupled to triple quadrupole mass spectrometry (LC-MS/MS) in a complementary manner (33, 34).

### 2.3 Instrumental analysis

#### 2.3.1 CG-MS/MS

A total of 129 out of 353 compounds were analyzed by GC-MS/MS. To that end, an Agilent 7890B gas chromatographer was used, along with an Agilent 7693 automatic sampler and a tandem-coupled Agilent 7010 mass spectrometer (Agilent Technologies, Santa Clara, California, USA). The injection volume was 1.5μL and two fused (5%-phenyl)-methylpolysiloxane silica ultra-inert capillary columns (Agilent J&W HP-5MS, Agilent Technologies) were used for chromatographic separation. Both columns were 15 m long, had an internal diameter of 0.25 mm and a film thickness of 0.25 μm. The selected carrier gas was helium (99.9999% purity), which was pumped in constant flow. Nitrogen (99.9999% purity, Linde, Dublin, Ireland) was used as a collision gas. With regards to the oven temperature, the program was as follows: 1.8 min at 80°C; increase of 40°C/min until reaching 170°C; increase of 10°C/min until 310°C; and hold at 310°C for 3 min. The multiple reaction monitoring (MRM) mode was used in runs of 21.05 min, divided in 24-time segments.

#### 2.3.2 LC-MS/MS

A total of 224 out of 353 analytes were quantified using an Agilent 1290 ultrahigh-performance liquid chromatographer (UHPLC), coupled in tandem with an Agilent 6460 mass spectrometer (Agilent Technologies, Palo Alto, USA). An InfinityLab Poroshell 120 (100mm of length, 2.1 mm of thickness and a particle diameter of 2.7 μm) was the column of choice for chromatographic separation. The mobile phases A and B were composed of ultrapure water (2 mM ammonium acetate; 0.1% FA) and MeOH (2 mM ammonium acetate), respectively. A volume of 8 μL was injected at a flow rate of 0.4 mL/min, with an oven temperature of 50°C. Each run lasted for 18 min. The dynamic MRM (dMRM) mode was selected to operate the mass spectrometer. A nitrogen generator NGMs-1 (Atlas Copco, Stockholm, Sweden) was used to produce nitrogen as a drying and desolvation gas. As a collision gas, Nitrogen 6.0 (99.9999% purity, Linde, Dublin, Ireland) was used.

The optimal operating conditions of the mass spectrometer analyses for both GC-MS/MS and LC-MS/MS have been described in previous work and can be found in **Supplementary Table S1** in the **Supplementary Material** (33).

### 2.4 Quality of analysis and quality control

The limits of quantification (LOQs) for the analysis of these analytes have been previously established (33). Briefly, LOQs were determined by performing recovery assays, being the lowest concentrations, whose accuracy ranged from 70% to 120% and the standard deviation was lower than 20% and can be referenced in **Supplementary Table S1**. These criteria were stablished in the SANTE/12682/2019 guidance definition (35). Every 25 vials, an internal QC sample was included, which was obtained by spiking fetal bovine serum (FBS; Lonza, Basel, Switzerland) at 5 ng/mL with all analytes and subsequently processing as the rest of serum samples. QCs were accepted when the percentages of recovery ranged between 70% and 120% and the standard deviation was lower than 20%, according to the SANTE 12682/2019 recommendations (35).

### 2.5 Statistical analysis

PASW Statistics v19.0 (SPSS Inc., Chicago, Illinois, USA) was used to perform statistical analyses. Chi-squared tests, Wilcoxon test and Mann-Whitney U tests were conducted for comparisons before and after surgery of sociodemographic variables, for detection rates, and concentrations, respectively. The Kolmogorov–Smirnov test for normality was used to check the distributions of sociodemographic variables and POPs. A Student’s t test was used to analyze the variation of total lipids. Bivariate correlation Kendall’s τ test was performed to study the influence of sociodemographic characteristics on the differences in serum levels of POPs. The results were reported as medians and interquartile ranges. Concentration values below the LOD were not included in the statistical analysis. Those values between the LOD and the LOQ were only taken into account to determine the detection rates. To study concentration changes before and after surgery, half of the LOQ was taken if the compound had not been quantified at that time point.

Serum total cholesterol and triglyceride concentrations were determined enzymatically and used for the lipid adjustment of results (36, 37). POP concentrations were individually corrected for total lipids (TL) by dividing the raw POP concentration by TL and are expressed in nanograms of analyte per gram of lipid weight (ng/g lw).

The level of statistical significance was set at 0.05 and all tests were two tailed.

## 3 Results

Before surgery, mean weight was 129.7 kg which translates into a mean BMI of 47.7 kg/m^2^. After surgery (mean follow-up of 534 ± 24.1), Mann-Whitney U tests revealed that these parameters were significantly reduced and reached 83.6 kg (ρ < 0.001) and 31.0 kg/m^2^ (ρ < 0.001), respectively (**Table 2**). Thus, a mean weight loss of 45.28 kg was observed. According to the BMI, all patients had at least class II obesity and 84.7% had class III obesity at the beginning of the study. Bariatric surgery resulted in an important decrease in the frequency of class III obesity and 21.4% of the patients achieved normal weight (**Table 2**). Remarkably, only one patient failed to lose weight during this period, showing an increase of 0.6 kg. Since body weight was affected by the intervention, Mann-Whitney U tests also showed a significant reduction in total lipids in blood from 6.6 mg/mL to 5.9 mg/mL (ρ < 0.001), which involves a mean reduction of 0.668 mg/mL.

**Table 2:** Changes observed after surgery in the patients included in the study (mean follow-up (days): 534 ± 24.1).

Variables	Before surgery	After surgery	*P-value*
	(n = 56)	(n = 55)	
Weight (kg)			
Mean ± SD	129.7 ± 24.2	83.6 ± 19.2	<0.001^†^
Median	127.5	83.6	
BMI (kg/m^2^)*			
Mean ± SD	47.7 ± 6.5	31.0 ± 6.2	<0.001^†^
Median	48.3	30.7	
Normal weight	0	12 (21.4)	<0.001^††^
Overweight	0	14 (25.0)	
Obesity (grade I)	0	14 (25.0)	
Obesity (grade II)	9 (15.3)	11 (19.6)	
Obesity (grade III)	50 (84.7)	5 (8.9)	
TBWL (kg)			
Mean ± SD		45.3 ± 20.7	NA
Median		43.3	NA
%EWL		65.4	NA
Smoking habit^#^			
Yes	10 (16.9)	14 (23.7)	0.229^††^
No	46 (78.0)	41 (69.5)	
Number of cigarettes			
Mean ± SD	11.6 ± 6.3	15.0 ± 7.6	0.355^†^
Median	11.0	12.0	
Total lipids (mg/mL)			
Mean ± SD	6.6 ± 0.9	5.9 ± 1.1	0.001^†††^
Median	6.5	6.1	

SD, standard deviation; BMI, body mass index; TBWL, total body weight loss; %EWL, percentage of excess body weight loss; NA, not applicable.

*Normal weight: 18.5 – 24.99, overweight: 25.0 – 29.99, obesity (grade I): 30 – 34.99, obesity (grade II): 35 – 39.99, obesity (grade III): ≥40. Missed follow-up: 3 patients.

Missed data before surgery: 3 patients; missed data after surgery: 4 patients.

^†^Mann-Whitney U test.

^††^Chi-squared test.

^†††^Student’s t-test.

A total of 353 compounds, including POPs, pesticides, pharmaceuticals, and rodenticides, were analyzed. Among them, 55 POPs –including 16 polycyclic aromatic hydrocarbons (PAH), 8 polybrominated diphenyl ethers (PBDE), 18 polychlorinated biphenyls (PCB) congeners and 13 organochlorine pesticides (OCP)- were included (**Supplementary Table S1**). The results shown below refer solely and exclusively to the compounds detected and quantified in the series.

Among the 55 POPs analyzed, dichlorodiphenyldichloroethilene (p,p’-DDE), hexachlorobenzene (HCB), β-hexachlorocyclohexane (β-HCH), PCB-138, PCB-153, PCB-180 and phenanthrene were identified in serum both before and after surgery (**Table 3**). Of note, p,p’-DDE displayed the highest frequency of detection before and after surgery, being detected in 69.6% and 96.2% of the patients, respectively. Changes were also observed in the detection rates of HCB (from 5.4% to 44.2%), β-HCH (from 8.9% to 59.9%), PCB-138 (from 16.1% to 75.0%), PCB-153 (from 10.7% to 61.5%), PCB-180 (from 10.7% to 73.1%) and phenanthrene (from 3.6% to 67.3%), with ρ<0.001 in all cases (**Table 3**). Certain OCPs, such as dichlorodiphenyldichloroethane (p,p’-DDD) and dichlorodiphenyltrichloroethane (p,p’-DDT), were detected in less than 5% of the series prior the surgery and were not present afterwards. Interestingly, naphthalene was only detected after the intervention, being observed in 71.2% of the patients. Other non-POP compounds were detected either before or after surgery. For instance, both frequency of detection and concentration of 2-phenylphenol changed during the follow-up, from 14.3% to 57.7% (ρ < 0.001) and from 1.2 ppb to 0.36 ppb (ρ = 0.029). This was not the case for acetaminophen, whose frequency of detection remained the same, even though mean concentration increased from 3.9 ppb to 9.9 ppb after surgery (**Table 3**). Lastly, fenitrothion and flucythrinate II were only detected after surgery, being present in 42.3% and 11.5% of the cohort, respectively.

**Table 3:** Serum concentrations of detected substances before and after surgery. Detection rates and concentrations [expressed in median (percentile 25^th^—percentile 75^th^)] are included, in parts per billion (ng/mL) and, in the case of POPs, in ng/g lw.

	Before surgery (n = 56)^1^			After surgery (n = 52)^2^				
	Detection rate [N, (%)]	Concentration (ppb)	Concentration (ng/g lw)	Detection rate [N, (%)]	Concentration (ppb)	Concentration (ng/g lw)	P value^#^	P value^†^
p,p’-DDD	1 (1.8)	0.16	24.2	0	—	—	NA	NA
p,p´-DDE	39 (69.6)	1.1 (0.4 – 3.8)	210.3 (68.0 – 534.1)	50 (96.2)***	2.1 (1.0 – 9.0)	476.0 (180.5 – 1365.9)	0.009	0.003
p,p´-DDT	2 (3.6)	1.2 (1.1 – 1.3)	176.3 (156.6 – 195.9)	0	—	—	NA	NA
HCB	3 (5.4)	0.60 (0.3 – 0.9)	76.8 (41.5 – 136.9)	23 (44.2)***	0.56 (0.4 – 1.1)	79.9 (65.3 – 208.7)	0.688	0.316
β-HCH	5 (8.9)	0.85 (0.3 – 1.1)	128.2 (37.3 – 146.3)	27 (51.9)***	0.56 (0.3 – 1.4)	96.1 (67.0 – 231.9)	0.736	0.421
Naphthalene	0	—	—	37 (71.2)***	1.0 (0.9 – 1.1)	178.8 (155.7 – 228.1)	NA	NA
PCB-138	9 (16.1)	0.13 (0.1 – 0.2)	19.8 (17.7 – 23.4)	39 (75.0)***	0.21 (0.2 – 0.3)	35.2 (26.2 – 53.7)	0.002	<0.001
PCB-153	6 (10.7)	0.17 (0.1 – 0.2)	23.4 (17.6 – 32.6)	32 (61.5)***	0.27 (0.2 – 0.4)	44.3 (32.4 – 65.5)	0.020	0.003
PCB-180	6 (10.7)	0.13 (0.1 – 0.2)	19.5 (17.9 – 22.6)	38 (73.1)***	0.26 (0.2 – 0.4)	43.8 (29.9 – 65.6)	0.012	0.001
Phenanthrene	2 (3.6)	0.63 (0.5 – 0.8)	103.5 (83.5 – 123.5)	35 (67.3)***	0.37 (0.3 – 0.5)	62.3 (46.9 – 87.1)	0.075	0.093
2-Phenylphenol	8 (14.3)	1.2 (0.2 – 2.0)		30 (57.7)***	0.36 (0.8 – 8.6)		0.029	
Fenitrothion	0	—		22 (42.3)***	2.0 (1.7 – 2.4)		NA	
Flucythrinate I	0	—		2 (3.8)	0.24 (0.2 – 0.3)		NA	
Flucythrinate II	0	—		6 (11.5)*	0.16 (0.1 – 0.2)		NA	
Carbofuran	2 (3.6)	1.8 (0.3 – 3.2)		0	—		NA	
Acetaminophen	24 (42.9)	3.9 (3.0 — 15.1)		17 (32.7)	9.9 (7.4 – 18.4)		0.024	
Diclofenac	1 (1.8)	3.7		1 (1.9)	2.5		0.317	
Ketoprophen	0	—		3 (5.8)	16.9 (7.4 – 30.9)		NA	
Levamisole	1 (1.8)	172.4		3 (5.8)	11.9 (1.9 – 16.4)		0.180	
Naproxen^	4 (7.1)	5.9 (1.4 – 12.1)		3 (5.8)	0.65 (0.03 – 4.0)		0.157	

POPs, persistent organic pollutants; NA, not applicable.

^1^No serum samples for three patients.

^2^No serum samples for seven patients.

*Comparison between detection rates before and after surgery (Chi-squared test). *** P < 0.001; * P < 0.05.

Comparison of serum concentrations of substances (ppb) before and after surgery (Mann-Whitney U test).

^†^Comparison of serum concentrations of substances (ng/g lw) before and after surgery (Mann-Whitney U test).

^Concentration expressed in mg/mL.

**Figure 1:**
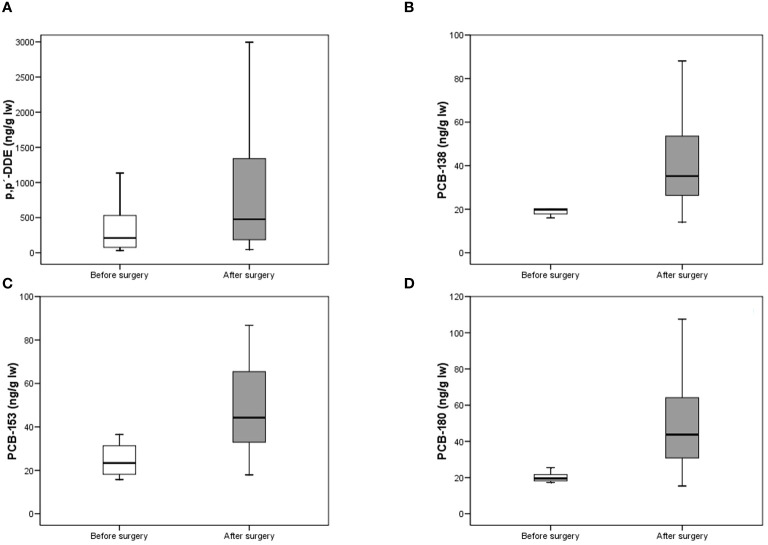
Concentrations (ng/g lw) of p,p’-DDE **(A)**, PCB-138 **(B)**, PCB-153 **(C)** and PCB-180 **(D)** before and after surgery (Mann-Whitney U test). The lines connect the medians, the boxes cover the 25th to 75th percentiles, and the minimal and maximal values are shown by the ends of the bars.

After surgery, serum levels of p,p’-DDE increased from 210.3 ng/g lw to 476.0 ng/g lw (ρ = 0.003). PCB-138 increased from 19.8 ng/g lw to 35.2 ng/g lw (ρ < 0.001). Concentrations also increased from 23.4 ng/g lw to 44.3 ng/g lw in the case of PCB-153 (ρ = 0.003) and from 19.5 ng/g lw to 43.8 ng/g lw in the case of PCB-180 (ρ = 0.001) (**Table 3**, **Figure 1**). As for naphthalene, serum levels after surgery were 178.8 ng/g lw. With regards to phenanthrene, no significant changes in concentration were observed. We replicated the analyses using the Wilcoxon test, observing the same trend: ρ < 0.001, ρ = 0.028, ρ = 0.043 and ρ = 0.043 for p,p’-DDE and the PCB congeners -138, -153, and -180, respectively (Data not shown). No other significant differences were observed (**Table 3**). We wanted to explore which serum lipid parameters might be related to the observed changes in POPs levels. Before surgery, we did not observe any significant correlation between POPs concentrations and serum cholesterol, HDL, LDL or triglyceride levels (Data not shown). After surgery we observed a negative correlation between the concentration of HCB and HDL, so that the higher the HCB concentration, the lower the HDL concentration (τ = -0.576; ρ = 0.009). The same trend was observed between naphthalene and cholesterol (τ = -0.529; ρ = 0.001), HDL (τ = -0.438; ρ = 0.005), LDL (τ = -0.467; ρ = 0.003), and triglycerides (τ = -0.382; ρ = 0.013). Finally, a similar negative correlation was observed between phenanthrene and triglycerides (τ = -0.469; ρ = 0.005). No other significant correlations were detected.

Following the surgical intervention, 12 patients reduced their weight to achieve normal values, while 16 remained with obesity type II-III (**Table 2**). We did not observe concentration differences of any of the analyzed substances between both groups (Mann-Whitney U test, ρ > 0.05 in all cases). In order to determine whether the increase observed in serum concentrations of POPs was associated with other variables included in the study, a bivariate correlation test was performed (**Table 4**). By analyzing the whole series, the elevation of naphthalene levels positively correlated with weight loss (τ = 0.317; ρ = 0.006), while a negative association with the increment of total lipids was observed (τ = -0.395; ρ = 0.001). However, segregation by sex revealed a different trend in women than in men. On one hand, the increase of naphthalene in females followed the pattern of the cohort when treated as a whole (for weight loss, τ = 0.351 and ρ = 0.012; for total lipids, τ = -0.439 and ρ = 0.002). In males, on the other, this increase was not associated with weight loss or increase of total lipids, but with time after surgery (τ = 0.661; ρ = 0.005). In the case of phenanthrene, a negative association with the increment of total lipids was observed both in the whole series (τ = -0.458; ρ < 0.001) and in women (τ = 0.529; ρ < 0.001), but not in men (**Table 4**). Finally, a positive association between the increasing levels of PCB-138 and weight loss was shown (τ = 0.223; ρ = 0.036). Nonetheless, such association was not observed when the series was segregated by sex (**Table 4**). The analyses regarding %EWL only showed a significant correlation with PCB-138 in the complete series (τ = 0.250; ρ = 0.025), with no significant differences observed when segmented by gender (Data not shown). No other correlations between POP concentrations and other sociodemographic variables were found.

**Table 4:** Bivariate correlations between serum POPs concentration in relation to time after surgery, weight loss and total lipid increase.

Substance		Whole series			Females			Males		
		Time^1^	Lost weight	Δ TL	Time^1^	Lost weight	Δ TL	Time^1^	Lost weight	Δ TL
Δ p,p’-DDD	*Kendall’s Tau*	NA	NA	NA	NA	NA	NA	NA	NA	NA
	*P value*	—	—	—	—	—	—	—	—	—
Δ p,p´-DDE	*Kendall’s Tau*	0.025	0.073	0.027	0.026	0.036	-0.007	0.044	0.143	0.124
	*P value*	0.801	0.433	0.766	0.824	0.744	0.946	0.826	0.477	0.520
Δ p,p´-DDT	*Kendall’s Tau*	NA	NA	NA	NA	NA	NA	NA	NA	NA
	*P value*	—	—	—	—	—	—	—	—	—
Δ HCB	*Kendall’s Tau*	-0.096	0.275	-0.047	-0.262	0.228	-0.094	0.400	0.600	0.000
	*P value*	0.525	0.059	0.747	0.129	0.172	0.575	0.327	0.142	1.000
Δ β-HCH	*Kendall’s Tau*	NA	0.400	0.000	NA	0.400	0.000	NA	NA	NA
	*P value*	—	0.327	1.000	—	0.327	1.000	—	—	—
Δ Naphthalene	*Kendall’s Tau*	0.127	0.317	-0.395	-0.069	0.351	-0.439	0.661	0.309	-0.309
	*P value*	0.286	**0.006**	**0.001**	0.637	**0.012**	**0.002**	**0.005**	0.186	0.186
Δ PCB-138	*Kendall’s Tau*	0.032	0.233	-0.079	-0.136	0.233	-0.104	0.404	0.200	-0.030
	*P value*	0.783	**0.036**	0.477	0.332	0.082	0.440	0.086	0.392	0.891
Δ PCB-153	*Kendall’s Tau*	0.076	0.044	-0.048	-0.048	0.074	-0.026	0.404	-0.055	-0.127
	*P value*	0.547	0.721	0.698	0.762	0.632	0.865	0.086	0.815	0.586
Δ PCB-180	*Kendall’s Tau*	-0.108	0.109	-0.102	-0.249	0.180	-0.101	0.220	-0.018	0.127
	*P value*	0.352	0.327	0.364	0.077	0.179	0.452	0.349	0.938	0.586
Δ Phenanthrene	*Kendall’s Tau*	0.029	0.141	-0.458	-0.028	0.105	-0.529	0.294	0.273	-0.200
	*P value*	0.812	0.233	**<0.001**	0.853	0.472	**<0.001**	0.212	0.243	0.392

NA, not applicable; Δ TL, total lipids after surgery minus the concentration before surgery.

Δ Serum concentration of substances after surgery minus the concentration before surgery.

^1^Time between surgery and post-surgery (days).

Bold values indicate the significant results.

## 4 Discussion

The present study has demonstrated a significant increase in serum POPs levels associated with a substantial decrease in body weight following bariatric surgery. While age is indeed the primary demographic factor associated with increased levels of these substances, with a median of 532 days between both determinations, it is unlikely to be the underlying reason for the observed changes. Firstly, the levels of these substances have been decreasing over the decades (38, 39). Secondly, it has been observed that a decrease of 5 kilograms or more within a short period (1 year) is associated with an increase in POPs concentrations (38, 39). Lastly, recent studies have observed that patients undergoing bariatric surgery experience a significant increase in the concentrations of these substances in their bodies (40). In this regard, it is important to highlight that we did not observe significant changes in POPs levels due to the time elapsed between both measurements (Kendall’s τ test, ρ > 0.05 in all cases; Data not shown). However, to ascertain that any observed increases are attributable to rapid weight loss rather than solely accumulation over time, it would be interesting to compare the present findings with those of an intervention-free control group. Multiple control groups should be studied, comprising a lean group without intervention, an obese group without intervention, an obese group with gradual weight loss through lifestyle modifications, and an obese group with rapid weight loss through alternative methods such as lipectomy or pharmaceuticals. This approach would contribute to understanding the role of rapid weight loss in relation to the presence of these substances, especially when we do not observe significant differences between those patients who, after the surgical intervention, reduced their weight to normal values compared to those who remained with obesity type II-III. This suggests that the initial burden of pollutants, together with other demographic variables such as age and sex, significantly contribute to modulating the levels of these substances in the body.

To date, bariatric surgery is still the most effective intervention to treat obesity. In this study, we observed a mean weight loss of 45.28 kg, accounting for a 63.9% of excess weight loss (EWL), which allowed 21.4% of the patients to reach normal weight. This is in line with previous publications, in which patients managed to lose over 30 kg in one year (41, 42). It has been reported that maximum weight loss is achieved two years after surgery (43). Thus, differences in weight loss might be explained by the fact that patients were followed up for a longer period, namely 534 ± 24 days.

It was previously reported that p,p’-DDE, HCB, β-HCH, PCB-138 and PCB-180 were detected in more than half of the obese, elderly Canarian population (PREDIMED-plus) (18). In the present cohort, detection rates displayed by all POPs before surgery were lower detection rates than those observed in previous studies. However, they increased after surgery, being similar to those observed in the PREDIMED-plus for p,p’-DDE and even higher for PCB-138. Increases in the detection rates of circulating POPs after rapid weight loss have already been described (18, 41, 44) and it highlights the toxicological role of the adipose tissue, which is thought to sequester lipophilic xenobiotic molecules, thereby preventing other cell types from their deleterious effect (45).

Dichlorodiphenyldichloroethilene is a metabolite that results from the dehydrohalogenation of the organochlorine pesticide p,p’-DDT. Even though it was banned in most countries in the last decades of the 20^th^ century, p,p’-DDT, p,p’-DDE and p,p’-DDD are still present in the population worldwide (18, 41). In this cohort, p,p’-DDE was the most frequently detected POP and showed the highest serum levels both before and after surgery, with detection rates of 69.6% and 96.2% and concentrations of 210.3 ng/g lw and 476.0 ng/g lw, respectively. This contrasts with results from the PREDIMED-plus, in which a detection rate of 95.4% and a mean concentration of 697.4 ng/g lw were determined. BMI was stated as a predictor of p,p’-DDE concentration (18). Nonetheless, levels of p,p’-DDE observed in this study are generally lower, despite the fact that mean BMI was much higher before surgery. This results might be explained by differences in age between these two cohorts (66.4 vs 43.4 years), given that a positive correlation between age and serum levels of POPs has been demonstrated by our group (18). Considering that DDT was banned in Spain in the 1970s (46), older individuals were probably subject to direct exposure during pregnancy and early life, which might have led to higher serum levels of DDT metabolites in the adulthood. Yet, p,p’-DDE is still the most prevalent POP in the Canary islands regardless the mean age of the participants, probably due to intensive use, transplacental transfer during pregnancy (21) and bioaccumulation through the trophic chain (19).

A completely different scenario was observed in the case of HCB and β-HCH, whose detection rates increased after surgery but no concentration increment was observed. Conversely, previous studies on the relation between bariatric surgery and serum POPs showed an increase in levels of both OCPs (41). Increase in HCB and β-HCH levels after weight loss has been also reported in obese non-operated individuals (18, 47, 48). HCB and β-HCH are highly persistent (49), being the β-HCH the most resistant to degradation and the most prevalent in the environment (Srivastava, 50). Due to the mean age of the patients, direct exposure to these toxicants is not expected. Thus, dietary intake might have played an important role in their accumulation in the body due to biomagnification, given that they are especially susceptible to atmospheric deposition on the soil (51). More profound knowledge on diet and lifestyle may be necessary to fully comprehend the different routes of exposure.

With regards to the PCBs, they are industrial halogenated chemicals that were widely used as lubricants and coolants in electronic devices until their application was banned in the last decades of the 20^th^ century. More than 20 years later, numerous PCB congeners are still present in most populations and have been detected in blood and breast milk (52, 53), being the congeners 138, 153 and 180 the most frequently detected in the Canary islands (18, 39). In this study, these congeners were detected before and after surgery and displayed an increase in both detection rate and concentration. It has been stablished that PCB exposure takes place mainly via dietary intake and indoor air inhalation (54). However, differences in pharmacokinetics and degradation patterns hamper the determination of the primary composition of manufacture mixtures and, consequently, their origin (55). Remarkably, bivariate correlation tests revealed a significant positive association between PCB-138 serum levels and weight loss. This suggests a possible contribution of the adipose tissue by acting as an endogenous source of exposure in conditions of enhanced lypolysis (45). However, other variables must be considered when interpreting the present results. It is important to highlight the close relationship between serum levels of certain POPs (such as PCBs) and body composition. In this regard, it has been observed that visceral fat releases a greater amount of PCBs than subcutaneous fat in individuals subjected to strict weight loss diets, a finding that could not be replicated in patients undergoing bariatric surgery (56). Moreover, a significant negative correlation has been observed between BMI, waist circumference, fat mass percentage, total and subcutaneous abdominal adipose tissue, and serum PCB levels (57). Although our results support previous observations by other authors, it is necessary to understand the pharmacokinetics of these compounds to comprehend the consequences of fat tissue mobilization, especially when it occurs rapidly.

Polycyclic aromatic hydrocarbons are not POPs *per se*. Although they are semipersistent, their constant, massive release into the atmosphere has led them to be included in this category. Here, we report a substantial increase in the detection rate of both naphthalene and phenanthrene after surgery. To our knowledge, this is the first time that increases in detection rates and serum levels of PAHs after weight loss are described. Naphthalene and phenanthrene are petrogenic-origin volatile compounds that can be suspended in both indoor and outdoor air (58, 59), which suggests that transport from industrialized north African regions may contribute to exposure. Dietary intake via plant products and processed food is also considered an important source of exposure to PAHs (60, 61). Nonetheless, these factors might not fully explain such changes in the prevalence of these contaminants. We hypothesize that sequestration in scarcely metabolically active white adipose tissue, which constitutes a hallmark of obesity pathophysiology, could protect PAHs from degradation, thereby allowing them to accumulate. The observed concentration increase in women positively correlates with weight loss for naphthalene, and negatively correlates with variation in total lipids for both naphthalene and phenanthrene, thereby supporting this hypothesis. In that sense, further research in the mechanisms of interplay between blood and adipose tissue is required.

Given the health risks that obesity poses for the individuals, it is undeniable that bariatric surgery is the most effective treatment to prevent complications associated with this disease, thus constituting a potent tool to alleviate the healthcare system (13). This fact is particularly important in the Canarian archipelago, where obesity, diabetes and metabolic syndrome are highly prevalent (62). Nevertheless, such increase of serum levels of POPs after bariatric surgery-induced rapid weight loss should not be ignored, especially considering that physical exercise, as a weight control strategy, has been proposed as an effective approach to reduce the adverse effects of POPs in situations of rapid weight loss (63). In this regard, the benefits of physical exercise — healthier lifestyle, better vascular health, or better intestinal microecological balance— should be complemented by its ability to reduce the burden of POPs and their adverse effects (64). Therefore, beyond the outcome of bariatric surgery, physical exercise should complement obesity treatment, not only in mitigating the action of contaminants but also due to its enormous health benefits.

These contaminants have the capability to alter molecular, metabolic and endocrine pathways, leading to the development of diverse pathologies (65). For instance, PCBs and OCPs are molecules with endocrine-disrupting and neurotoxic potential whose exposure is associated with elevation of inflammatory markers, cardiovascular disorders, breast and prostate cancer and endometriosis (27, 49, 55, 66). Strong associations between carcinogenesis and PAH exposure have also been demonstrated and additive effects in mixtures of PAHs have been suggested (67, 68). Particularly, naphthalene and phenanthrene, which were highly prevalent in this series after surgery, are correlated with erythrocyte disruption and cardiovascular disease, respectively (69). Furthermore, synergistic effects have been described in certain POPs (70), although the role of exposure-related pathogenesis remains unclear. Based on our results, it would be expected that the literature would report associations between bariatric surgery and pathologies related to POPs, such as neurotoxicity or cancer. While it is true that an association between bariatric surgery and certain neurological pathologies has been observed (71), some authors attribute it to nutritional deficiencies (72, 73) while others do not (74). Therefore, it cannot be ruled out that certain toxins mobilized as a result of weight loss may play a role. Regarding cancer, the literature shows that bariatric surgery reduces the incidence of both hormone-dependent and non-hormone-dependent cancers (75–77). Overall, even though the risk-benefit ratio of bariatric surgery is positive, and policies have proven effective on lowering the environmental burden of POPs, we believe that strong follow-up, legislative and biomonitoring measures are required to prevent the development of exposure-associated pathologies.

## 5 Conclusion

Bariatric surgery-induced weight loss led to a significant increase of detection rates and serum levels of p,p’-DDE, HCB, β-HCH and PCB congeners 138, 153 and 180. Detection rates of PAHs, namely naphthalene and phenanthrene, also increased after surgery. Sex differences were found with respect to the correlation between concentration of naphthalene, phenanthrene and PCB-138 and weight loss, total lipids and time after surgery. To our knowledge, the effect of bariatric surgery on the concentration of POPs had not previously been assessed in Spain. Moreover, this is the first time that presence of circulating PAHs after surgery is recorded. The role of adipose tissue as a source of POP exposure and the potential effects of the release of these compounds into the circulatory system are not clear. Thus, toxicokinetics of POPs, as their effects on peripheral tissues, and the mechanisms underlying adipocyte-blood transfer, require further investigation.

## Data availability statement

The original contributions presented in the study are included in the article/**Supplementary Material**. Further inquiries can be directed to the corresponding author.

## Ethics statement

The studies involving humans were approved by Clinical Research Ethics Committee of the CHUIMI (Complejo Hospitalario Universitario Insular Materno Infantil): ethical approval code 2024-093-728, on June 26th, 2014. The studies were conducted in accordance with the local legislation and institutional requirements. The participants provided their written informed consent to participate in this study.

## Author contributions

BVD-G: Data curation, Writing – review & editing, Methodology. ÁR-L: Writing – review & editing. LAH-H: Data curation, Formal analysis, Supervision, Writing – review & editing. LS-M: Conceptualization, Writing – review & editing. IB-C: Data curation, Methodology, Writing – review & editing. AA-D: Methodology, Writing – review & editing. OPL: Funding acquisition, Supervision, Writing – review & editing. EH-G: Data curation, Writing – review & editing. JC-T: Data curation, Writing – review & editing. JRH-H: Data curation, Writing – review & editing. PF-V: Methodology, Supervision, Writing – original draft.
