# Supplementary material for: Effect of bariatric surgery in the body burden of persistent and non-persistent pollutants: longitudinal study in a cohort of morbidly obese patients
Source: Front Endocrinol (Lausanne). 2024 Jul 22;15:1412261. doi: 10.3389/fendo.2024.1412261 (PMC11298429; doi:10.3389/fendo.2024.1412261)
Supplement: Supplementary file 1 [file Table_1.docx]

**Table S1. List compounds analyzed with their LOQ, category of use, analysis technique, retention time and mass spectrometry conditions.**

| **Compound** | **Category ^a^** | **Technique** ^b^ | **Retention time (min)** | **Polarity** | **Quantification** | | **Confirmation** | | **Fragmentor** | **LOQ (ng/mL)** |
| --- | --- | --- | --- | --- | --- | --- | --- | --- | --- | --- |
|  |  |  |  |  | **MRM transition (m/z)** | **CE (eV)** | **MRM transition (m/z)** | **CE (eV)** |  |  |
| 2-Phenylphenol | P | GC | 6.28 | positive | 169.0 ➔ 115.0 | 30 | 169.0 ➔ 141.0 | 15 | 70 | 0.6 |
| 4,4′-Dichlorobenzophenone (metabolite of dicofol) | P | GC | 10 | positive | 250.0 ➔ 139.0 | 15 | 250.0 ➔ 215.0 | 5 | 70 | 0.3 |
| Abamectine | P | LC | 10.98 | positive | 890.5 ➔ 567.1 | 10 | 895.5 ➔ 751.4 | 45 | 160 | 2.5 |
| Acenaphthene | POP | GC | 5.93 | positive | 153.0 ➔ 152.0 | 25 | 153.0 ➔ 151.0 | 35 | 70 | 0.3 |
| Acenaphtylene | POP | GC | 6.14 | positive | 152.0 ➔ 151.0 | 25 | 152.0 ➔ 126.0 | 30 | 70 | 0.6 |
| Acephate | P | LC | 1.65 | positive | 184.0 ➔ 143.0 | 15 | 143.0 ➔ 95.0 | 15 | 70 | 2.5 |
| Acetaminophen (Paracetemol) | M | LC | 2.76 | positive | 152.1 ➔ 65.0 | 40 | 152.1 ➔ 93.0 | 20 | 150 | 1.25 |
| Acetamiprid | P | LC | 4.44 | positive | 223.1 ➔ 126.0 | 27 | 223.1 ➔ 90.0 | 45 | 140 | 0.3 |
| Acrinathrin | P | LC | 10.71 | positive | 559.0 ➔ 208.0 | 10 | 559.0 ➔ 181.0 | 30 | 70 | 0.6 |
| Albendazole | M | LC | 7.26 | positive | 266.1 ➔ 234.1 | 16 | 266.1 ➔ 191.0 | 32 | 155 | 0.15 |
| Aldicarb | P | LC | 5.17 | positive | 208.0 ➔ 116.0 | 10 | 116.0 ➔ 89.1 | 4 | 100 | 0.15 |
| Aldicarb-sulfone | P | LC | 2.8 | positive | 240.1 ➔ 76.0 | 16 | 223.1 ➔ 86.1 | 13 | 75 | 0.6 |
| Aldicarb-sulfoxide | P | LC | 2.75 | positive | 207.1 ➔ 131.9 | 10 | 207.1 ➔ 89.1 | 10 | 86 | 1.25 |
| Aldrin | POP | GC | 9.89 | positive | 255.0 ➔ 220.0 | 25 | 263.0 ➔ 228.0 | 10 | 70 | 0.3 |
| Anthracene | POP | GC | 8.4 | positive | 178.0 ➔ 176.0 | 35 | 178.0 ➔ 152.0 | 30 | 70 | 0.6 |
| Atrazine | P | LC | 6.77 | positive | 216.0 ➔ 173.9 | 15 | 216.0 ➔ 103.8 | 30 | 130 | 0.15 |
| Azinphos-methyl | P | LC | 7.28 | positive | 318.0 ➔ 132.1 | 8 | 340.0 ➔ 160.0 | 10 | 60 | 0.15 |
| Azoxystrobin | P | LC | 7.59 | positive | 404.1 ➔ 372.1 | 8 | 404.1 ➔ 344.1 | 24 | 110 | 0.15 |
| BDE-28 | POP | GC | 12.23 | positive | 406.0 ➔ 246.0 | 20 | 406.0 ➔ 167.0 | 25 | 70 | 0.15 |
| BDE-47 | POP | GC | 14.32 | positive | 326.0 ➔ 138.0 | 45 | 484.0 ➔324.0 | 25 | 70 | 0.3 |
| BDE-85 | POP | GC | 17.1 | positive | 564.0 ➔ 404.0 | 25 | 566.0 ➔ 406.0 | 25 | 70 | 0.3 |
| BDE-99 | POP | GC | 16.28 | positive | 566.0 ➔ 406.0 | 25 | 564.0 ➔ 404.0 | 30 | 70 | 0.3 |
| BDE-100 | POP | GC | 15.86 | positive | 566.0 ➔ 406.0 | 25 | 564.0 ➔ 404.0 | 25 | 70 | 0.3 |
| BDE-153 | POP | GC | 18.06 | positive | 644.0 ➔ 484.0 | 25 | 486.0 ➔ 377.0 | 30 | 70 | 0.15 |
| BDE-154 | POP | GC | 17.5 | positive | 644.0 ➔ 484.0 | 25 | 486.0 ➔ 377.0 | 30 | 70 | 0.3 |
| BDE-183 | POP | GC | 20.14 | positive | 561.6 ➔ 454.7 | 40 | 563.6 ➔ 454.7 | 40 | 70 | 0.15 |
| Benalaxyl | P | LC | 8.98 | positive | 326.2 ➔ 148.0 | 20 | 326.2 ➔ 208.0 | 12 | 90 | 0.15 |
| Bendiocarb | P | LC | 5.92 | positive | 224.1 ➔ 166.9 | 8 | 224.2 ➔ 108.9 | 15 | 120 | 0.3 |
| Bendiocarb metabolite (2, 2-dimethylbenzo-1, 3-dioxol-4-ol) | P | GC | 4.83 | positive | 166.0 ➔ 151.0 | 10 | 166.0 ➔ 126.0 | 20 | 70 | 2.5 |
| Benfuracarb | P | LC | 9.73 | positive | 411.2 ➔ 190.0 | 13 | 411.2 ➔ 252.0 | 15 | 110 | 1.25 |
| Benzo[a]anthracene | POP | GC | 13.88 | positive | 228.0 ➔ 226.0 | 40 | 228.0 ➔ 202.0 | 35 | 70 | 0.15 |
| Benzo[a]pyrene | POP | GC | 16.91 | positive | 252.0 ➔ 250.0 | 45 | 252.0 ➔ 248.0 | 60 | 70 | 0.15 |
| Benzo[b]fluoranthene | POP | GC | 16.27 | positive | 252.0 ➔ 248.0 | 60 | 252.0 ➔ 226.0 | 35 | 70 | 0.3 |
| Benzo[ghi]perylene | POP | GC | 19.65 | positive | 276.0 ➔ 274.0 | 50 | 276.0 ➔ 272.0 | 60 | 70 | 0.15 |
| Benzo[k]fluoranthene | POP | GC | 16.3 | positive | 252.0 ➔ 250.0 | 45 | 252.0 ➔ 224.0 | 40 | 70 | 0.3 |
| Bifenthrin | P | GC | 13.89 | positive | 440.0 ➔ 181.0 | 5 | 440.0 ➔ 165.0 | 60 | 94 | 0.15 |
| Bitertanol | P | LC | 9.22 | positive | 338.2 ➔ 70.0 | 4 | 338.2 ➔ 269.2 | 5 | 100 | 0.3 |
| Boscalid (formerly nicobifen) | P | GC | 16.55 | positive | 3434.0 ➔ 272.0 | 30 | 343.0 ➔ 140.0 | 45 | 100 | 0.15 |
| Brodifacoum | AR | LC | 10.64 | negative | 521.3 ➔ 79.0 | 50 | 523.3 ➔ 135.0 | 45 | 220 | 0.3 |
| Bromadiolone | AR | LC | 9.7 | negative | 525.3 ➔ 250.0 | 40 | 527.3 ➔ 250.0 | 40 | 200 | 0.3 |
| Bromopropylate | P | GC | 13.87 | positive | 341.0 ➔ 183.0 | 15 | 341.0 ➔ 157.0 | 45 | 70 | 0.15 |
| Bromuconazole (two isomers) | P | GC | 13.81 | positive | 295.0 ➔ 173.0 | 10 | 295.0 ➔ 175.0 | 10 | 70 | 0.3 |
| Bupirimate | P | LC | 8.4 | positive | 273.0 ➔ 108.0 | 15 | 273.0 ➔ 193.0 | 5 | 70 | 0.3 |
| Buprofezin | P | LC | 9.88 | positive | 306.1 ➔ 201.0 | 12 | 306.1 ➔ 116.0 | 12 | 140 | 0.15 |
| Cadusafos (ebufos) | P | LC | 9.4 | positive | 271.1 ➔ 159.0 | 16 | 271.1 ➔ 131.0 | 22 | 100 | 0.3 |
| Carbaryl | P | LC | 6.24 | positive | 202.1 ➔ 145.1 | 4 | 202.1 ➔ 127.1 | 28 | 95 | 0.15 |
| Carbendazim (azole) | P | LC | 3.4 | positive | 192.1 ➔ 160.1 | 4 | 202.1 ➔ 127.1 | 28 | 90 | 0.3 |
| Carbofuran | P | LC | 5.95 | positive | 222.1 ➔ 123.1 | 20 | 222.1 ➔ 165.1 | 30 | 80 | 0.15 |
| Carbofuran-3-hydroxy | P | LC | 4.27 | positive | 238.1 ➔ 163.1 | 10 | 238.1 ➔ 181.1 | 10 | 110 | 0.6 |
| Cefuroxima axetil (two isomers) | M | LC | 5.4 | positive | 533.0 ➔ 447.0 | 15 | 533.0 ➔ 386.0 | 20 | 160 | 0.3 |
| Chloramphenicol | M | LC | 4.62 | negative | 321.0 ➔ 152.1 | 4 | 323.0 ➔ 152.1 | 4 | 113 | 2.5 |
| Chlorantraniliprole | P | LC | 7.33 | positive | 483.9 ➔ 452.9 | 16 | 483.9 ➔ 285.9 | 8 | 105 | 0.3 |
| Chlorfenapyr | P | GC | 12.01 | positive | 247.0 ➔ 200.0 | 30 | 247.0 ➔ 227.0 | 15 | 70 | 0.6 |
| Chlorfenvinphos | P | LC | 9.08 | positive | 361.1 ➔ 98.9 | 34 | 358.9 ➔ 155.1 | 8 | 105 | 0.3 |
| Chlorobenzilate | P | GC | 12.14 | positive | 251.0 ➔ 111.0 | 40 | 251.0 ➔ 139.0 | 15 | 70 | 0.15 |
| Chlorophacinone | AR | LC | 8.75 | negative | 373.2 ➔ 201.0 | 20 | 375.2 ➔ 203.0 | 20 | 160 | 5 |
| Chlorpropham | P | GC | 7.12 | positive | 213.0 ➔ 127.0 | 15 | 153.0 ➔ 90.0 | 25 | 70 | 0.15 |
| Chlorpyrifos | P | GC | 9.93 | positive | 314.0 ➔ 258.0 | 15 | 314.0 ➔ 286.0 | 5 | 70 | 0.15 |
| Chlorpyrifos methyl | P | GC | 9.12 | positive | 286.0 ➔ 93.0 | 25 | 286.0 ➔ 271.0 | 15 | 70 | 0.15 |
| Chlorthal dimethyl | POP | GC | 10.03 | positive | 300.9 ➔ 166.9 | 55 | 300.9 ➔ 222.9 | 25 | 70 | 0.15 |
| Chrysene | M | GC | 13.95 | positive | 228.0 ➔ 226.0 | 40 | 228.0 ➔ 227.0 | 25 | 70 | 0.3 |
| Clindamycin | P | LC | 5.65 | positive | 425.2 ➔ 126.1 | 20 | 425.2 ➔ 377.2 | 20 | 150 | 1.25 |
| Clofentezine | P | LC | 9.2 | positive | 303.1 ➔ 138.0 | 12 | 303.1 ➔ 102.0 | 40 | 120 | 0.3 |
| Clothianidin | M | LC | 3.9 | positive | 250.0 ➔ 169.0 | 8 | 250.0 ➔ 131.9 | 8 | 100 | 1.25 |
| Cortiscosterone 21 acetate | P | LC | 7.89 | positive | 389.1 ➔ 329.0 | 13 | 389.1 ➔ 371.0 | 13 | 80 | 1.25 |
| Coumachlor | P | LC | 8.6 | positive | 343.1 ➔ 162.8 | 15 | 342.1 ➔ 285.0 | 15 | 120 | 0.6 |
| Coumaphos | AR | LC | 8.99 | positive | 363.0 ➔ 227.0 | 30 | 363.0 ➔ 306.9 | 15 | 120 | 0.6 |
| Coumatetralyl | P | LC | 8.26 | negative | 291.1 ➔ 141.0 | 30 | 291.1 ➔ 247.0 | 20 | 140 | 0.6 |
| Cyazofamid | P | LC | 8.48 | positive | 325.0 ➔ 108.0 | 20 | 325.0 ➔ 261.1 | 15 | 90 | 0.6 |
| Cyflufenamid | P | LC | 9.19 | positive | 413.1 ➔ 223.1 | 33 | 413.1 ➔ 295.1 | 23 | 70 | 0.3 |
| Cyfluthrin (sum of four isomers) | P | GC | 16.21 | positive | 226.0 ➔ 206.0 | 25 | 198.9 ➔ 170.1 | 25 | 70 | 2.5 |
| Cyhalothrin (lambda isomer) | P | LC | 10.48 | positive | 181.1 ➔ 152.1 | 10 | 181.1 -> 127.1 | 46 | 70 | 2.5 |
| Cymoxanil | P | LC | 4.7 | positive | 199.1 ➔ 128.0 | 4 | 199.1 ➔ 110.9 | 12 | 90 | 1.25 |
| Cypermethrin (sum of four isomers) | P | GC | 16.54 | positive | 163.0 ➔ 109.0 | 20 | 163.0 ➔ 127.0 | 5 | 70 | 1.25 |
| Cyproconazole (two isomers) | P | LC | 8.14 | positive | 292.2 ➔ 70.2 | 18 | 292.2 ➔ 125.1 | 24 | 100 | 0.3 |
| Cyprodinil | P | LC | 8.57 | positive | 226.0 ➔ 93.0 | 33 | 226.0 ➔ 108 | 25 | 100 | 0.6 |
| Cyromazine | M | LC | 1.23 | positive | 167.1 ➔ 85.0 | 16 | 167.1 ➔ 125.0 | 20 | 120 | 2.5 |
| Danofloxacin | P | LC | 3.53 | positive | 358.2 ➔ 340.1 | 20 | 358.2 ➔ 82.1 | 50 | 159 | 2.5 |
| Dazomet | POP | GC | 7.81 | positive | 161.9 ➔ 44.0 | 28 | 161.9 ➔ 89.0 | 5 | 70 | 0.3 |
| Deltamethrin | POP | LC | 10.64 | positive | 523.0 ➔ 281.0 | 10 | 523.0 ➔ 506.0 | 5 | 100 | 1.25 |
| Demeton-S-methyl | POP | LC | 6 | positive | 230.9 ➔ 88.9 | 5 | 230.9 ➔ 61.0 | 30 | 50 | 0.15 |
| Demeton-S-methyl-sulfone (Dioxydemeton) | P | LC | 3.3 | positive | 263.0 ➔ 169.0 | 24 | 263.0 ➔ 109.0 | 12 | 120 | 0.6 |
| Dexamethasone | P | LC | 7.16 | positive | 393.2 ➔ 373.2 | 2 | 393.2 ➔ 355.2 | 6 | 103 | 1.25 |
| Diazinon | P | GC | 8.28 | positive | 137.1 ➔ 54.0 | 20 | 304.0 ➔ 179.0 | 15 | 70 | 0.15 |
| Dibenzo[a,h]anthracene | M | GC | 19.18 | positive | 278.0 ➔ 276.0 | 40 | 278.0 ➔ 250.0 | 60 | 70 | 0.15 |
| Dichlorodiphenyldichloroethane (p,p′ DDD) | P | GC | 12.32 | positive | 235.0 ➔ 165.0 | 20 | 235.0 ➔ 199.0 | 15 | 70 | 0.15 |
| Dichlorodiphenyldichloroethylene (p,p′ DDE) | POP | GC | 11.58 | positive | 318.0 ➔ 176.0 | 60 | 318.0 ➔ 248.0 | 30 | 70 | 0.3 |
| Dichlorodiphenyltrichloroethane (p,p′ DDT) | P | GC | 12.98 | positive | 235.0 ➔ 165.0 | 40 | 235.0 ➔ 199.0 | 15 | 70 | 1.25 |
| Diclofenac | P | LC | 8.73 | positive | 296.0 ➔ 215.1 | 16 | 296.0 ➔ 214.1 | 48 | 103 | 2.5 |
| Dicloran | M | GC | 7.8 | positive | 206.0 ➔ 176.0 | 10 | 206.0 ➔ 148.0 | 25 | 70 | 0.6 |
| Dichlorvos | POP | LC | 5.79 | positive | 221.0 ➔ 79.1 | 28 | 221.0 ➔ 109.1 | 16 | 105 | 0.6 |
| Dieldrin | P | GC | 11.67 | positive | 263.0 ➔ 228.0 | 15 | 277.0 ➔ 241.0 | 15 | 70 | 1.25 |
| Diethathyl ethyl | P | LC | 8.73 | positive | 312.2 ➔ 238.1 | 15 | 312.2 ➔ 162.0 | 30 | 120 | 0.15 |
| Diethofencarb | AR | LC | 7.59 | positive | 268.2 ➔ 226.1 | 5 | 268.2 ➔ 152.0 | 20 | 110 | 0.15 |
| Difenacoum | P | LC | 10.25 | negative | 443.2 ➔ 135.0 | 40 | 443.2 ➔ 293.0 | 35 | 200 | 0.3 |
| Difenoconazole | AR | LC | 9.41 | positive | 406.1 ➔ 250.9 | 28 | 406.1 ➔ 337.0 | 16 | 176 | 0.3 |
| Difethialone | M | LC | 10.8 | negative | 537.3 ➔ 79.0 | 50 | 537.3 ➔ 151.0 | 45 | 220 | 0.6 |
| Difloxacin | P | LC | 3.85 | positive | 400.2 ➔ 382.1 | 20 | 400.2 ➔ 356.1 | 16 | 149 | 2.5 |
| Diflubenzuron | P | LC | 8.63 | positive | 311.0 ➔ 158.0 | 8 | 311.0 ➔ 141.0 | 32 | 90 | 1.25 |
| Diflufenican | P | GC | 13.27 | positive | 394.0 ➔ 266.0 | 10 | 266.0 -> 246.0 | 10 | 70 | 0.15 |
| Dimethenamid-P (and its R-isomer) | P | LC | 7.72 | positive | 276.1 ➔ 244.1 | 10 | 276.1 ➔ 168.1 | 20 | 125 | 0.15 |
| Dimethoate | P | LC | 4.2 | positive | 230.0 ➔ 125.0 | 16 | 230.0 ➔ 198.8 | 20 | 70 | 0.6 |
| Dimethomorph (two isomers) | P | LC | 7.87 | positive | 388.1 ➔ 301.1 | 20 | 388.1 ➔ 165.1 | 32 | 180 | 0.3 |
| Dimethylphenylsulfamide (DMSA, metabolite of dichlofluanid) | P | LC | 5.24 | positive | 201.1 ➔ 92.1 | 15 | 201.1 ➔ 137.1 | 5 | 100 | 1.25 |
| Diniconazole-M | P | GC | 12.27 | positive | 326.1 ➔ 70.0 | 15 | 328.1 ➔ 70.0 | 15 | 70 | 0.3 |
| Dinocap | AR | LC | 10.43 | negative | 295.4 ➔ 208.9 | 30 | 295.4 ➔ 193.0 | 35 | 150 | 1.25 |
| Diphacinone | P | LC | 8.45 | negative | 339.1 ➔ 167.0 | 25 | 339.1 ➔ 145.0 | 20 | 170 | 5 |
| Diphenylamine | P | GC | 6.97 | positive | 168.0 ➔ 167.2 | 15 | 169.0 ➔ 66.0 | 15 | 70 | 0.3 |
| N,N-dimethylformamidine (DMF, metabolite of amitraz) | P | LC | 5.48 | positive | 150.1 ➔ 77.0 | 40 | 149.9 ➔ 105.8 | 30 | 100 | 1.25 |
| Dodine | P | LC | 9.1 | positive | 228.3 ➔ 43.0 | 40 | 228.3 ➔ 57.0 | 25 | 150 | 0.6 |
| Endosulfan alfa | P | GC | 11.21 | positive | 241.0 ➔ 206.0 | 15 | 195.0 ➔ 160.0 | 10 | 70 | 0.3 |
| Endosulfan beta | P | GC | 12.22 | positive | 241.0 ➔ 206.0 | 15 | 195.0 ➔ 159.0 | 15 | 70 | 0.3 |
| Endosulfan sulfate | P | GC | 12.97 | positive | 270.0 ➔ 235.0 | 15 | 387.0 ➔ 289.0 | 5 | 70 | 0.3 |
| Endrin | P | GC | 12.05 | positive | 263.0 ➔ 193.0 | 35 | 245.0 ➔ 173.0 | 25 | 70 | 1.25 |
| Enrofloxacin | P | LC | 3.62 | positive | 360.2 ➔ 316.1 | 16 | 360.2 ➔ 245.1 | 28 | 144 | 2.5 |
| EPN | POP | GC | 13.9 | positive | 157.0 ➔ 63.0 | 10 | 157.0 ➔ 110.0 | 15 | 70 | 0.3 |
| Epoxiconazole | M | LC | 8.47 | positive | 330.0 ➔ 120.9 | 24 | 330.1 ➔ 100.9 | 50 | 120 | 0.3 |
| Eprinomectin | P | LC | 10.83 | positive | 878.5 ➔ 186.0 | 15 | 936.5 ➔ 490.4 | 60 | 160 | 2.5 |
| Eritromicin | P | LC | 6.83 | positive | 734.5 ➔ 158.1 | 32 | 734.5 ➔ 576.3 | 16 | 172 | 0.3 |
| Esfenvalerate | M | GC | 17.56 | positive | 167.1 ➔ 125.1 | 15 | 167.1 ➔ 89.1 | 45 | 70 | 1.25 |
| Ethion (diethion) | M | LC | 10.02 | positive | 385.0 ➔ 199.0 | 5 | 385.0 ➔ 171.0 | 10 | 100 | 0.15 |
| Ethirimol | P | LC | 4.8 | positive | 210.2 ➔ 140.1 | 20 | 210.2 ➔ 98.1 | 28 | 160 | 0.6 |
| Ethofumesate | P | GC | 9.59 | positive | 286.0 ➔ 207.0 | 5 | 286.0 ➔ 161.0 | 20 | 70 | 0.15 |
| Ethoprophos | P | LC | 8.41 | positive | 243.1 ➔ 97.0 | 30 | 243.1 ➔ 130.9 | 15 | 90 | 0.3 |
| Etofenprox | P | GC | 16.75 | positive | 163.0 ➔ 107.0 | 20 | 163.0 ➔ 135.0 | 10 | 70 | 0.3 |
| Etoxazole | P | LC | 10.33 | positive | 360.1 ➔ 141.0 | 26 | 360.1 ➔ 304.0 | 16 | 160 | 0.15 |
| Famoxadone | P | LC | 9.05 | positive | 392.1 ➔ 330.9 | 5 | 392.2 ➔ 238.1 | 12 | 110 | 0.6 |
| Fenamidone | P | LC | 7.73 | positive | 392.1 ➔ 330.9 | 5 | 392.1 ➔ 238.1 | 12 | 110 | 0.3 |
| Fenamiphos | P | LC | 8.64 | positive | 304.1 ➔ 217.1 | 20 | 304.1 ➔ 202.0 | 36 | 120 | 0.3 |
| Fenamiphos sulfone | P | LC | 6.17 | positive | 336.1 ➔ 188.0 | 31 | 336.1 ➔ 266.0 | 23 | 120 | 0.3 |
| Fenamiphos sulfoxide | P | LC | 5.92 | positive | 320.1 ➔ 233.0 | 20 | 320.1 ➔ 108.1 | 44 | 120 | 1.25 |
| Fenarimol | P | GC | 15.04 | positive | 139.0 ➔ 75.0 | 30 | 139.0 ➔ 111.0 | 15 | 70 | 0.15 |
| Fenazaquin | P | LC | 10.75 | positive | 307.2 ➔ 57.1 | 25 | 307.2 ➔ 161.1 | 16 | 90 | 0.15 |
| Fenbendazole | P | LC | 8.09 | positive | 300.1 ➔ 268.1 | 20 | 300.1 ➔ 159.0 | 36 | 156 | 0.15 |
| Fenbuconazole | P | GC | 16.18 | positive | 198.0 ➔ 102.0 | 30 | 198.0 ➔ 78.0 | 30 | 70 | 0.3 |
| Fenbutatin oxide | M | LC | 11.61 | positive | 519.0 ➔ 197.0 | 55 | 517.3 ➔ 194.9 | 60 | 180 | 0.3 |
| Fenhexamid | P | LC | 8.35 | positive | 302.1 ➔ 97.1 | 20 | 302.1 ➔ 55.1 | 40 | 130 | 1.25 |
| Fenitrothion | P | GC | 9.57 | positive | 277.0 ➔ 109.0 | 15 | 277.0 ➔ 125.0 | 15 | 70 | 1.25 |
| Fenoxycarb | P | LC | 8.69 | positive | 302.1 ➔ 88.0 | 20 | 302.1 ➔ 116.1 | 10 | 110 | 0.3 |
| Fenpropathrin | P | LC | 14 | positive | 367.2 ➔ 125.0 | 16 | 350.0 ➔ 125.0 | 16 | 72 | 0.6 |
| Fenpropidin | P | LC | 7.25 | positive | 274.3 ➔ 147.0 | 30 | 274.3 ➔ 86.0 | 25 | 170 | 0.15 |
| Fenpropimorph | P | LC | 7.51 | positive | 304.3 ➔ 147.1 | 30 | 304.3 ➔ 130.0 | 25 | 120 | 0.15 |
| Fenpyroximate | P | LC | 10.49 | positive | 422.2 ➔ 366.2 | 12 | 422.2 ➔ 135.0 | 36 | 160 | 0.3 |
| Fenthion | P | GC | 9.89 | positive | 278.0 ➔ 109.0 | 15 | 278.0 -> 125.0 | 20 | 70 | 0.15 |
| Fenthion oxon | P | LC | 7.33 | positive | 263.1 ➔ 231.2 | 16 | 263.1 ➔ 216.0 | 24 | 120 | 0.3 |
| Fenthion oxon sulfone | P | LC | 4.5 | positive | 295.0 ➔ 217.0 | 15 | 295.0 ➔ 104.2 | 24 | 110 | 0.6 |
| Fenthion oxon sulfoxide | P | LC | 4.25 | positive | 279.0 ➔ 264.2 | 20 | 279.0 ➔ 104.1 | 28 | 110 | 0.6 |
| Fenthion sulfone | P | LC | 6.39 | positive | 311.0 ➔ 125.0 | 22 | 311.0 ➔ 109.0 | 28 | 140 | 1.25 |
| Fenthion sulfoxide | P | LC | 6.16 | positive | 295.0 ➔ 108.9 | 30 | 295.0 ➔ 280.0 | 18 | 140 | 0.6 |
| Fenvalerate | P | GC | 17.37 | positive | 167.0 ➔ 125.1 | 22 | 167.0 ➔ 89.0 | 30 | 70 | 1.25 |
| Fipronil | P | LC | 10.63 | negative | 435.0 ➔ 330.0 | 12 | 435.0 ➔ 249.9 | 26 | 116 | 0.15 |
| Fipronil sulfide | P | GC | 10.55 | positive | 351.0 ➔ 255.0 | 20 | 420.0 ➔ 351.0 | 25 | 70 | 1.25 |
| Flocoumafen | P | LC | 10.35 | negative | 541.3 ➔ 382.0 | 25 | 541.3 ➔ 161.0 | 40 | 230 | 0.3 |
| Fluazinam | P | LC | 10 | negative | 462.9 ➔ 416.0 | 10 | 462.9 ➔ 398.0 | 9 | 140 | 0.6 |
| Flubendiamide | AR | LC | 8.79 | positive | 408.0 ➔ 274.0 | 15 | 408.0 ➔ 256.0 | 30 | 120 | 1.25 |
| Flucythrinate (two isomers) | P | GC | 16.68 | positive | 156.9 ➔ 107.1 | 15 | 199.1 ➔ 107.1 | 25 | 70 | 0.15 |
| Fludioxonil | P | GC | 11.52 | positive | 248.0 ➔ 127.0 | 30 | 248.1 ➔ 182.1 | 10 | 70 | 0.15 |
| Flufenoxuron | P | LC | 10.34 | positive | 489.1 ➔ 158.0 | 20 | 489.1 ➔ 140.9 | 56 | 110 | 0.6 |
| Flumequine | P | LC | 6.12 | positive | 262.1 ➔ 244.0 | 16 | 262.1 ➔ 202.0 | 32 | 116 | 0.3 |
| Flunixin | P | LC | 8.1 | positive | 297.1 ➔ 279.1 | 24 | 297.1 ➔ 264.1 | 32 | 141 | 0.6 |
| Fluopyram | M | GC | 10.62 | positive | 173.0 ➔ 95.0 | 35 | 223.0 ➔ 196.0 | 40 | 70 | 0.15 |
| Fluoranthene | M | GC | 10.68 | positive | 202.0 ➔ 201.0 | 27 | 202.0 ➔ 152.0 | 42 | 70 | 0.15 |
| Fluorene | P | GC | 6.8 | positive | 165.0 ➔ 163.0 | 40 | 165.0 ➔ 139.0 | 30 | 70 | 0.6 |
| Fluquinconazole | POP | GC | 15.81 | positive | 340.0 ➔ 298.0 | 15 | 340.0 ➔ 286.0 | 25 | 70 | 0.3 |
| Flusilazole | POP | LC | 8.65 | positive | 316.1 ➔ 247.1 | 15 | 316.1 ➔ 165.0 | 20 | 160 | 0.3 |
| Flutolanil | P | LC | 7.93 | positive | 324.1 ➔ 262.1 | 16 | 324.1 ➔ 242.1 | 24 | 130 | 0.15 |
| Flutriafol | P | GC | 11.26 | positive | 219.0 ➔ 95.0 | 35 | 219.0 ➔ 123.0 | 15 | 70 | 0.3 |
| Fluvalinate tau | P | GC | 17.57 | positive | 250.1 ➔ 55.1 | 30 | 252.0 ➔ 200.0 | 20 | 70 | 1.25 |
| Fonofos | P | GC | 8.24 | positive | 246.0 ➔ 109.0 | 15 | 246.0 ➔ 237.0 | 5 | 70 | 0.3 |
| Formetanate | P | LC | 1.77 | positive | 222.1 ➔ 165.1 | 12 | 222.1 ➔ 46.2 | 28 | 105 | 2.5 |
| Fosthiazate | P | LC | 6.53 | positive | 284.0 ➔ 104.0 | 20 | 284.0 ➔ 227.8 | 8 | 90 | 0.15 |
| Heptachlor | P | GC | 9.3 | positive | 272.0 ➔ 237.0 | 15 | 274.0 ➔ 239.0 | 15 | 70 | 0.15 |
| Hexachlorobencene | P | GC | 7.76 | positive | 284.0 ➔ 214.0 | 40 | 284.0 ➔ 249.0 | 25 | 70 | 0.3 |
| Hexachlorocyclohexane (alpha) | POP | GC | 7.63 | positive | 219.0 ➔ 109.0 | 10 | 219.0 ➔ 183.0 | 10 | 70 | 0.3 |
| Hexachlorocyclohexane (beta) | POP | GC | 8.03 | positive | 219.0 ➔ 109.0 | 40 | 219.0 ➔ 183.0 | 5 | 70 | 0.3 |
| Hexachlorocyclohexane (delta) | POP | GC | 8.5 | positive | 219.0 ➔ 109.0 | 45 | 219.0 ➔ 183.0 | 5 | 70 | 0.6 |
| Hexaclorocyclohexane (gamma, lindane) | POP | GC | 8.13 | positive | 291.0 ➔ 109.0 | 40 | 219.0 ➔183.0 | 10 | 70 | 1.25 |
| Hexaconazole (two isomers) | POP | LC | 8.49 | positive | 314.1 ➔ 70.1 | 20 | 316.1 ➔ 70.1 | 20 | 95 | 0.3 |
| Hexaflumuron | P | LC | 9.57 | negative | 458.8 ➔ 439.0 | 8 | 458.8 ➔ 175.0 | 30 | 100 | 0.6 |
| Hexythiazox | P | LC | 10.18 | positive | 353.1 ➔ 227.9 | 8 | 353.1 ➔ 168.1 | 24 | 120 | 0.15 |
| Imazalil (enilconazole) | P | LC | 6.53 | positive | 297.1 ➔ 159.0 | 20 | 297.1 ➔ 69.1 | 18 | 100 | 0.15 |
| Imidacloprid | P | LC | 4.1 | positive | 256.0 ➔ 175.0 | 12 | 256.0 ➔ 209.0 | 12 | 110 | 0.6 |
| Indeno [1,2,3-cd] pyrene | P | GC | 19.11 | positive | 276.0 ➔ 274.0 | 50 | 276.0 ➔ 272.0 | 60 | 70 | 0.3 |
| Indoxacarb | POP | LC | 9.47 | positive | 528.1 ➔ 293.1 | 10 | 528.1 ➔ 202.8 | 48 | 140 | 0.6 |
| Iprodione | P | GC | 13.67 | positive | 314.0 ➔ 56.0 | 20 | 314.0 ➔ 245.0 | 10 | 70 | 1.25 |
| Iprovalicarb | P | LC | 8.2 | positive | 321.2 ➔ 119.0 | 15 | 321.2 ➔ 202.9 | 20 | 110 | 0.3 |
| Isocarbophos | P | GC | 10.37 | positive | 230.0 ➔ 155.0 | 25 | 230.0 ➔ 198.0 | 10 | 70 | 0.3 |
| Isofenphos methyl | P | LC | 8.82 | positive | 199.0 ➔ 121.0 | 10 | 241.0 ➔ 121.0 | 25 | 70 | 0.15 |
| Isoprothiolane | P | GC | 11.45 | positive | 291.1 ➔ 189.0 | 30 | 291.1 ➔ 145.0 | 36 | 100 | 0.3 |
| Ivermectin B1a | P | LC | 11.5 | positive | 897.5 ➔ 753.5 | 50 | 897.5 ➔ 329.3 | 60 | 160 | 1.25 |
| Josamycin | M | LC | 7.48 | positive | 860.5 ➔ 173.9 | 40 | 860.5 ➔ 108.9 | 40 | 200 | 0.3 |
| Ketoprofen | M | LC | 7.34 | positive | 255.1 ➔ 209.1 | 8 | 255.1 ➔ 77.1 | 48 | 123 | 1.25 |
| Kresoxim methyl | M | LC | 8.8 | positive | 116.0 ➔ 89.0 | 15 | 206.0 ➔ 131.0 | 10 | 70 | 0.6 |
| Leptophos | P | GC | 14.58 | positive | 171.1 ➔ 77.1 | 15 | 377.0 ➔ 362.0 | 20 | 70 | 0.3 |
| Levamisole | P | LC | 3.19 | positive | 205.1 ➔ 178.1 | 20 | 205.1 ➔ 123.0 | 32 | 141 | 0.6 |
| Lincomycin | M | LC | 3.57 | positive | 407.2 ➔ 126.1 | 24 | 407.2 ➔ 359.2 | 16 | 150 | 1.25 |
| Linuron | M | LC | 7.56 | positive | 249.0 ➔ 160.1 | 20 | 249.0 ➔ 182.3 | 8 | 120 | 0.6 |
| Lufenuron | POP | LC | 10.04 | negative | 509.0 ➔ 339.0 | 5 | 509.0 ➔ 326.1 | 15 | 90 | 0.3 |
| Malaoxon | P | LC | 6.05 | positive | 315.1 ➔ 127.2 | 12 | 315.1 ➔ 99.1 | 36 | 120 | 0.15 |
| Malathion | P | LC | 7.95 | positive | 348.0 ➔ 126.7 | 15 | 348.0 ➔ 285.0 | 8 | 100 | 0.15 |
| Mandipropamid | P | LC | 7.9 | positive | 412.1 ➔ 328.1 | 8 | 412.1 ➔ 356.1 | 4 | 130 | 0.15 |
| Mebendazole | P | LC | 6.7 | positive | 296.1 ➔ 264.1 | 20 | 296.1 ➔ 77.0 | 48 | 151 | 0.3 |
| Mefenamic acid | P | LC | 9.5 | positive | 242.1 ➔ 209.1 | 28 | 242.1 ➔ 180.1 | 0 | 108 | 0.6 |
| Mefenoxam (metalaxyl-M) | M | LC | 6.97 | positive | 280.0 ➔ 220.0 | 10 | 280.0 ➔ 192.0 | 15 | 110 | 0.15 |
| Meloxicam | M | LC | 7.13 | positive | 352.5 ➔ 114.8 | 20 | 352.5 ➔ 140.8 | 20 | 130 | 0.6 |
| Mepanipyrim | P | GC | 11.14 | positive | 222.0 ➔ 221.0 | 15 | 222.0 ➔ 207.0 | 15 | 70 | 0.6 |
| Mepiquat | M | LC | 0.66 | positive | 114.0 ➔ 98.0 | 36 | 114.0 ➔ 70.0 | 45 | 100 | 0.6 |
| Metaflumizone | P | LC | 9.92 | negative | 505.0 ➔ 302.0 | 14 | 541.0 ➔ 302.0 | 20 | 90 | 0.3 |
| Metalaxyl | P | GC | 9.31 | positive | 234.0 ➔ 146.1 | 20 | 249.0 ➔ 146.0 | 20 | 70 | 0.15 |
| Metaldehyde | P | LC | 3.94 | positive | 194.1 ➔ 61.9 | 5 | 194.1 ➔ 106.0 | 5 | 50 | 1.25 |
| Metconazole | P | LC | 9.17 | positive | 320.1 ➔ 70.2 | 33 | 322.1 ➔ 70.2 | 24 | 250 | 0.15 |
| Methamidophos (two isomers) | P | LC | 1.18 | positive | 142.0 ➔ 94.0 | 12 | 142.0 ➔ 125.0 | 12 | 85 | 2.5 |
| Methidathion | P | LC | 7.13 | positive | 320.1 ➔ 144.8 | 8 | 320.1 ➔ 85.0 | 30 | 84 | 0.15 |
| Methiocarb | P | LC | 7.68 | positive | 226.1 ➔ 169.0 | 4 | 226.1 ➔ 121.1 | 12 | 90 | 0.3 |
| Methiocarb-sufone | P | LC | 4.52 | positive | 258.1 ➔ 201.1 | 8 | 258.1 ➔ 122.1 | 22 | 100 | 1.25 |
| Methiocarb-sulfoxide | P | LC | 4.03 | positive | 242.0 ➔ 185.0 | 22 | 242.0 ➔ 122.0 | 28 | 90 | 0.6 |
| Methomyl | P | LC | 3.22 | positive | 163.1 ➔ 88.0 | 5 | 163.0 ➔ 106.0 | 8 | 80 | 0.6 |
| Methoxyfenozide | P | LC | 8 | positive | 369.2 ➔ 149.0 | 10 | 369.2 ➔ 313.1 | 15 | 85 | 0.15 |
| Metoxychlor | P | GC | 13.98 | positive | 227.0 ➔ 141.0 | 20 | 227.0 ➔ 169.0 | 15 | 70 | 1.25 |
| Metrafenone | POP | LC | 9.29 | positive | 409.1 ➔ 209.1 | 8 | 411.1 ➔ 209.1 | 12 | 108 | 0.15 |
| Metronidazole | P | LC | 2.55 | positive | 172.1 ➔ 128.0 | 12 | 172.1 ➔ 82.1 | 24 | 98 | 1.25 |
| Mevinphos (phosdrin) | P | LC | 4.43 | positive | 225.0 ➔ 193.1 | 15 | 225.0 ➔ 127.0 | 12 | 65 | 1.25 |
| Mirex | M | GC | 14.8 | positive | 237.0 ➔ 143.0 | 30 | 274.0 ➔ 237.0 | 10 | 70 | 0.3 |
| Monocrotophos | P | LC | 3.3 | positive | 224.1 ➔ 126.8 | 12 | 224.1 ➔ 98.1 | 15 | 100 | 1.25 |
| Moxidectin | POP | LC | 11.26 | positive | 641.4 ➔ 529.2 | 5 | 641.4 ➔ 499.2 | 5 | 100 | 1.25 |
| Myclobutanil | P | LC | 8.1 | positive | 289.1 ➔ 70.1 | 16 | 289.1 ➔ 125.1 | 32 | 110 | 0.15 |
| N-(2,4-dimethylphenyl)-N′-methylformamidine (DMPF, metabolite of amitraz) | M | LC | 3.35 | positive | 163.1 ➔ 122.1 | 15 | 163.1 ➔ 107.1 | 15 | 100 | 2.5 |
| N,N-Dimethyl-N′-p-tolylsulphamide (DMST, metabolite of tolyfluanid) | P | LC | 6.09 | positive | 215.1 ➔ 106.1 | 10 | 215.1 ➔ 151.1 | 4 | 90 | 0.6 |
| Naphtalene | POP | GC | 4.45 | positive | 128.0 ➔ 127.0 | 15 | 128.0 ➔ 102.0 | 25 | 70 | 1.25 |
| Naproxen | M | LC | 7.59 | positive | 231.0 ➔ 185.0 | 10 | 231.1 ➔ 169.9 | 21 | 120 | 5 |
| Novobiocin | M | LC | 9.62 | positive | 613.2 ➔ 218.1 | 10 | 613.2 ➔ 396.1 | 10 | 150 | 2.5 |
| Nuarimol | P | GC | 13.24 | positive | 235.0 ➔ 139.0 | 15 | 235.0 ➔ 111.0 | 40 | 70 | 0.15 |
| Ofurace | P | LC | 12.75 | positive | 282.0 ➔ 159.9 | 20 | 282.0 ➔ 147.9 | 30 | 100 | 0.3 |
| Omethoate | P | LC | 2.85 | positive | 214.1 ➔ 124.8 | 22 | 214.1 ➔ 183.0 | 5 | 100 | 0.6 |
| Oxadixyl | P | LC | 5.46 | positive | 279.1 ➔ 219.2 | 5 | 279.1 ➔ 132.2 | 32 | 110 | 0.6 |
| Oxamyl | P | LC | 2.87 | positive | 237.1 ➔ 72.0 | 12 | 237.1 ➔ 90.0 | 5 | 70 | 0.3 |
| Oxfendazole | M | LC | 5.63 | positive | 316.1 ➔ 159.0 | 32 | 316.1 ➔ 191.1 | 16 | 166 | 0.3 |
| Oxolinic acid | P | LC | 5.06 | positive | 262.1 ➔ 216.0 | 32 | 262.1 ➔ 160.0 | 36 | 110 | 0.6 |
| Oxydemeton methyl | M | LC | 3 | positive | 247.0 ➔ 169.0 | 12 | 247.0 ➔ 109.0 | 24 | 100 | 0.6 |
| Oxyfluorfen | P | GC | 11.69 | positive | 252.0 ➔ 146.0 | 40 | 300.0 ➔ 223.0 | 15 | 70 | 0.3 |
| Paclobutrazol | P | LC | 11.05 | positive | 294.1 ➔ 70.1 | 16 | 294.1 ➔ 125.2 | 36 | 115 | 0.15 |
| Parathion methyl | P | GC | 9.12 | positive | 263.0 ➔ 109.0 | 15 | 263.0 ➔ 79.0 | 30 | 70 | 0.3 |
| PCB 28 | POP | GC | 9.02 | positive | 256.0 ➔ 186.0 | 25 | 256.0 ➔ 151.0 | 50 | 70 | 0.3 |
| PCB 52 | POP | GC | 9.58 | positive | 292.0 ➔ 222.0 | 25 | 292.0 ➔ 220.0 | 25 | 70 | 0.15 |
| PCB 77 | POP | GC | 11.74 | positive | 292.0 ➔ 220.0 | 25 | 292.0 ➔ 222.0 | 25 | 70 | 0.15 |
| PCB 81 | POP | GC | 11.57 | positive | 292.0 ➔ 220.0 | 25 | 292.0 ➔ 222.0 | 25 | 70 | 0.15 |
| PCB 101 | POP | GC | 11.08 | positive | 326.0 ➔ 256.0 | 30 | 328.0 ➔ 256.0 | 30 | 70 | 0.15 |
| PCB 105 | POP | GC | 12.67 | positive | 326.0 ➔ 256.0 | 30 | 328.0 ➔ 256.0 | 30 | 70 | 0.15 |
| PCB 114 | POP | GC | 12.39 | positive | 326.0 ➔ 256.0 | 30 | 328.0 ➔ 256.0 | 30 | 70 | 0.15 |
| PCB 118 | POP | GC | 12.19 | positive | 326.0 ➔ 256.0 | 30 | 328.0 ➔ 256.0 | 30 | 70 | 0.15 |
| PCB 123 | POP | GC | 12.14 | positive | 326.0 ➔ 256.0 | 30 | 328.0 ➔ 256.0 | 30 | 70 | 0.3 |
| PCB 126 | POP | GC | 13.23 | positive | 326.0 ➔ 256.0 | 30 | 328.0 ➔ 256.0 | 30 | 70 | 0.15 |
| PCB 138 | POP | GC | 13.07 | positive | 360.0 ➔ 290.0 | 25 | 360.0 ➔ 288.0 | 25 | 70 | 0.15 |
| PCB 153 | POP | GC | 12.58 | positive | 360.0 ➔ 290.0 | 25 | 360.0 ➔ 288.0 | 25 | 70 | 0.3 |
| PCB 156 | POP | GC | 13.97 | positive | 360.0 ➔ 290.0 | 25 | 360.0 ➔ 288.0 | 25 | 70 | 0.15 |
| PCB 157 | POP | GC | 14.07 | positive | 360.0 ➔ 290.0 | 25 | 360.0 ➔ 288.0 | 25 | 70 | 0.15 |
| PCB 167 | POP | GC | 13.56 | positive | 360.0 ➔ 290.0 | 25 | 360.0 ➔ 288.0 | 25 | 70 | 0.15 |
| PCB 169 | POP | GC | 14.62 | positive | 360.0 ➔ 290.0 | 25 | 360.0 ➔ 288.0 | 25 | 70 | 0.15 |
| PCB 180 | POP | GC | 14.25 | positive | 394.0 ➔ 324.0 | 30 | 394.0 ➔ 322.0 | 30 | 70 | 0.15 |
| PCB 189 | POP | GC | 15.26 | positive | 394.0 ➔ 324.0 | 30 | 394.0 ➔ 322.0 | 30 | 70 | 0.15 |
| Penconazole | P | GC | 10.51 | positive | 248.0 ➔ 157.0 | 30 | 248.0 ➔ 192.0 | 15 | 70 | 0.15 |
| Pencycuron | P | LC | 9.32 | positive | 329.1 ➔ 125.1 | 24 | 329.1 ➔ 217.9 | 12 | 160 | 0.15 |
| Pendimethalin | P | GC | 10.2 | positive | 252.0 ➔ 162.0 | 10 | 252.0 ➔ 191.0 | 5 | 70 | 0.3 |
| Penicilina V | M | LC | 6.48 | positive | 383.2 ➔ 159.9 | 10 | 383.2 ➔ 113.9 | 40 | 130 | 2.5 |
| Permethrin | P | GC | 15.7 | positive | 183.0 ➔ 128.0 | 15 | 183.1 ➔ 153.1 | 15 | 70 | 1.25 |
| Phenanthrene | POP | GC | 8.33 | positive | 178.0 ➔ 176.0 | 35 | 178.0 ➔ 152.0 | 28 | 70 | 0.3 |
| Phenylbutazone | M | LC | 8.24 | positive | 309.2 ➔ 160.2 | 20 | 309.2 ➔ 77.1 | 55 | 140 | 2.5 |
| Phosalone | P | LC | 9.2 | positive | 385.1 ➔ 182.0 | 20 | 385.1 ➔ 110.9 | 55 | 80 | 0.3 |
| Phosmet | P | LC | 7.35 | positive | 318.0 ➔ 159.9 | 16 | 318.0 ➔ 133.0 | 40 | 90 | 0.6 |
| Phosmet-oxon | P | LC | 5.39 | positive | 302.0 ➔ 160.0 | 10 | 302.0 ➔ 133.0 | 38 | 60 | 1.25 |
| Pirimicarb | P | LC | 5.31 | positive | 239.1 ➔ 72.1 | 20 | 239.1 ➔ 182.1 | 12 | 100 | 0.15 |
| Pirimicarb-desmethyl | P | LC | 3.6 | positive | 225.1 ➔ 168.1 | 8 | 225.1 ➔ 72.1 | 20 | 100 | 0.3 |
| Pirimiphos ethyl | P | GC | 10.26 | positive | 318.0 ➔ 166.0 | 15 | 318.0 ➔ 182.0 | 15 | 70 | 0.15 |
| Pirimiphos methyl | P | GC | 9.57 | positive | 306.1 ➔ 164.0 | 20 | 306.1 ➔ 108.1 | 32 | 100 | 0.15 |
| Prochloraz | P | LC | 9.11 | positive | 376.0 ➔ 308.0 | 10 | 376.0 ➔ 70.1 | 20 | 100 | 0.15 |
| Procymidone | P | GC | 10.8 | positive | 283.0 ➔ 67.0 | 40 | 283.0 ➔ 68.0 | 25 | 70 | 0.6 |
| Profenofos | P | LC | 9.75 | positive | 375.0 ➔ 305.0 | 20 | 373.0 ➔ 303.0 | 20 | 100 | 0.3 |
| Propamocarb | P | LC | 2.93 | positive | 189.2 ➔ 102.0 | 12 | 189.2 ➔ 144.0 | 8 | 110 | 0.6 |
| Propargite | P | LC | 10.35 | positive | 368.2 ➔ 231.1 | 4 | 368.2 ➔ 175.0 | 12 | 88 | 0.15 |
| Propiconazole | P | LC | 9.03 | positive | 342.0 ➔ 69.0 | 21 | 342.0 ➔ 159.0 | 39 | 90 | 0.6 |
| Propoxur | P | LC | 5.88 | positive | 210.1 ➔ 168.1 | 35 | 210.1 ➔ 65.1 | 40 | 70 | 0.3 |
| Propyzamide (pronamide) | P | LC | 7.94 | positive | 256.1 ➔ 190.0 | 16 | 256.1 ➔ 173.0 | 25 | 90 | 0.3 |
| Proquinazid | P | LC | 10.59 | positive | 288.0 ➔ 245.0 | 15 | 288.0 ➔ 217.0 | 30 | 70 | 0.15 |
| Prothioconazol | P | GC | 11.86 | positive | 186.0 ➔ 49.0 | 20 | 186.0 ➔ 53.0 | 25 | 70 | 0.15 |
| Prothiophos | P | GC | 11.46 | positive | 266.9 ➔ 221.0 | 35 | 162.0 ➔ 63.1 | 30 | 70 | 0.3 |
| Pymetrozine | P | LC | 2.8 | positive | 218.1 ➔ 105.0 | 20 | 218.1 ➔ 78.0 | 52 | 120 | 1.25 |
| Pyraclostrobin | P | LC | 9.15 | positive | 388.1 ➔ 193.8 | 8 | 388.1 ➔ 163.1 | 28 | 120 | 0.15 |
| Pyrazophos | P | LC | 9.23 | positive | 374.1 ➔ 222.1 | 23 | 374.1 ➔ 194.0 | 32 | 100 | 0.3 |
| Pyrene | POP | GC | 11.14 | positive | 202.0 ➔ 201.0 | 27 | 202.0 ➔ 200.0 | 45 | 70 | 0.3 |
| Pyridaben | P | LC | 10.75 | positive | 365.2 ➔ 309.0 | 8 | 309.1 ➔ 147.0 | 16 | 168 | 0.15 |
| Pyridaphenthion | P | LC | 8.11 | positive | 341.0 ➔ 189.0 | 22 | 341.0 ➔ 205.0 | 34 | 100 | 0.3 |
| Pyrimethanil | P | GC | 8.27 | positive | 198.0 ➔ 118.0 | 40 | 198.0 ➔ 158.0 | 20 | 70 | 0.15 |
| Pyriproxifen | P | LC | 10.08 | positive | 322.2 ➔ 96.0 | 12 | 322.2 ➔ 184.9 | 24 | 80 | 0.15 |
| Quinalfos | P | LC | 8.75 | positive | 299.1 ➔ 96.9 | 30 | 299.1 ➔147.1 | 20 | 130 | 0.3 |
| Quinoxyfen | P | LC | 10.13 | positive | 308.0 ➔ 197.0 | 32 | 308.2 ➔ 161.8 | 55 | 120 | 0.3 |
| Rifampicin | M | LC | 7.99 | positive | 823.5 ➔ 791.4 | 15 | 823.5 ➔ 399.1 | 25 | 160 | 2.5 |
| Rotenone | P | LC | 8.63 | positive | 395.1 ➔ 213.1 | 20 | 395.1 ➔ 192.1 | 25 | 150 | 0.6 |
| Roxithromycin | M | LC | 7.75 | positive | 838.5 ➔ 158.1 | 40 | 838.5 ➔ 116.1 | 55 | 200 | 0.6 |
| Simazine | P | LC | 5.87 | positive | 202.4 ➔ 68.1 | 30 | 202.4 ➔ 68.1 | 20 | 120 | 0.3 |
| Spinosad (two isomers) | P | LC | 9.19 | positive | 732.4 ➔ 142.0 | 22 | 732.4 ➔ 98.0 | 60 | 130 | 1.25 |
| Spiramycin (two isomers) | M | LC | 4.68 | positive | 439.1 ➔ 101.1 | 20 | 439.1 ➔ 88.0 | 50 | 70 | 2.5 |
| Spirodiclofen | P | LC | 10.51 | positive | 411.1 ➔ 71.2 | 15 | 411.1 ➔ 313.0 | 5 | 110 | 2.5 |
| Spiromesifen | P | LC | 10.28 | positive | 388.0 ➔ 273.0 | 25 | 273.0 ➔ 187.0 | 15 | 110 | 0.6 |
| Spiroxamine | P | LC | 7.7 | positive | 298.3 ➔ 144.1 | 16 | 298.3 ➔100.1 | 32 | 120 | 0.3 |
| Strychnine | AR | LC | 3.15 | positive | 335.1 ➔ 184.0 | 45 | 335.1 ➔ 156.0 | 40 | 105 | 0.6 |
| Sulfacetamide | M | LC | 2.12 | positive | 215.3 ➔ 155.9 | 10 | 215.3 ➔ 92.0 | 20 | 90 | 2.5 |
| Sulfachloropiridacine | M | LC | 3.75 | positive | 285.0 ➔ 156.0 | 12 | 285.0 ➔ 92.1 | 28 | 101 | 0.6 |
| Sulfadiacine | M | LC | 2.86 | positive | 251.0 ➔ 92.0 | 28 | 251.0 ➔ 156.0 | 12 | 111 | 1.25 |
| Sulfadimetoxine | M | LC | 4.81 | positive | 311.0 ➔ 92.0 | 32 | 311.0 ➔ 156.0 | 16 | 139 | 0.3 |
| Sulfadoxine | M | LC | 4.15 | positive | 311.1 ➔ 92.0 | 32 | 311.1 ➔ 156.0 | 16 | 126 | 0.3 |
| Sulfameracine | M | LC | 3.32 | positive | 265.0 ➔ 92.0 | 28 | 265.0 ➔ 156.0 | 12 | 126 | 0.6 |
| Sulfametacine | M | LC | 3.7 | positive | 279.1 ➔ 186.0 | 12 | 279.1 ➔ 92.0 | 32 | 134 | 0.6 |
| Sulfametizole | M | LC | 3.37 | positive | 271.0 ➔ 92.0 | 28 | 271.0 ➔ 155.9 | 8 | 103 | 1.25 |
| Sulfametoxazole | M | LC | 3.96 | positive | 254.0 ➔ 92.0 | 28 | 254.0 ➔ 156.0 | 12 | 111 | 0.6 |
| Sulfametoxipiridacine | M | LC | 3.77 | positive | 281.0 ➔ 155.9 | 12 | 281.0 ➔ 92.1 | 28 | 121 | 0.6 |
| Sulfamonomethoxine | M | LC | 4.03 | positive | 281.1 ➔ 156.0 | 14 | 281.1 ➔ 92.1 | 32 | 120 | 1.25 |
| Sulfapyridine | M | LC | 2.8 | positive | 250.0 ➔ 156.0 | 12 | 250.0 ➔ 92.0 | 28 | 126 | 1.25 |
| Sulfaquinoxaline | M | LC | 5 | positive | 301.0 ➔ 156.0 | 12 | 301.0 ➔ 92.1 | 32 | 159 | 0.6 |
| Sulfatiazole | M | LC | 3.05 | positive | 256.0 ➔ 92.0 | 28 | 256.0 ➔ 156.0 | 12 | 106 | 1.25 |
| Sulfisoxazole | M | LC | 4.14 | positive | 268.0 ➔ 156.0 | 8 | 268.0 ➔ 92.1 | 24 | 106 | 0.6 |
| Tebuconazole | P | LC | 8.92 | positive | 308.2 ➔ 70.2 | 22 | 308.2 ➔ 125.1 | 53 | 120 | 0.6 |
| Tebufenocide | P | LC | 8.67 | positive | 353.1 ➔ 132.9 | 22 | 353.1 ➔ 297.1 | 20 | 90 | 0.15 |
| Tebufenpyrad | P | LC | 14.07 | positive | 334.2 ➔ 117.0 | 47 | 334.2 ➔ 145.0 | 37 | 180 | 0.15 |
| Teflubenzuron | P | LC | 10 | negative | 379.0 ➔ 339.0 | 15 | 379.0 ➔ 196.0 | 25 | 100 | 1.25 |
| Tefluthrin | P | GC | 8.42 | positive | 177.0 ➔ 127.0 | 15 | 177.0 ➔ 87.0 | 15 | 70 | 0.15 |
| Telodrin (isobenzan) | P | GC | 10.15 | positive | 310.8 ➔ 240.8 | 25 | 310.8 ➔ 274.8 | 5 | 70 | 0.3 |
| Terbufos | P | GC | 8.15 | positive | 231.0 ➔ 97.0 | 20 | 231.0 ➔ 129.0 | 15 | 70 | 0.15 |
| Terbuthylazine | P | GC | 7.74 | positive | 214.0 ➔ 104.0 | 20 | 214.0 ➔ 132.0 | 10 | 70 | 0.15 |
| Tetrachlorvinphos | P | LC | 8.74 | positive | 367.0 ➔ 127.0 | 16 | 365.0 ➔ 127.0 | 16 | 110 | 0.3 |
| Tetraconazole | P | GC | 10.03 | positive | 336.0 ➔ 204.0 | 35 | 336.0 ➔ 218.0 | 20 | 70 | 0.3 |
| Tetradifon | P | GC | 14.36 | positive | 158.9 ➔ 111.0 | 20 | 354.0 ➔ 159.0 | 10 | 70 | 0.15 |
| Tetramethrin | P | GC | 13.81 | positive | 164.0 ➔ 77.0 | 30 | 164.0 ➔ 107.0 | 15 | 70 | 0.6 |
| Thiabendazole | P | LC | 3.9 | positive | 202.0 ➔ 175.0 | 24 | 202.0 ➔ 131.0 | 26 | 170 | 0.3 |
| Thiacloprid | P | LC | 4.81 | positive | 253.0 ➔ 126.0 | 16 | 253.0 ➔ 90.0 | 40 | 140 | 0.15 |
| Thiamethoxam | P | LC | 3.35 | positive | 292.0 ➔ 211.1 | 8 | 292.0 ➔ 132.0 | 22 | 80 | 2.5 |
| Thiophanate methyl | P | LC | 5.89 | positive | 343.0 ➔ 151.0 | 20 | 343.0 ➔ 93.0 | 46 | 90 | 0.3 |
| Tolclofos methyl | M | GC | 9.2 | positive | 265.0 ➔ 93.0 | 30 | 265.0 ➔ 220.0 | 25 | 70 | 0.3 |
| Tolfenamic acid | P | LC | 9.77 | negative | 260.0 ➔ 216.1 | 8 | 260.0 ➔ 35.1 | 20 | 108 | 1.25 |
| Triadimefon | M | LC | 8.05 | positive | 294.1 ➔ 69.3 | 20 | 294.1 ➔ 197.2 | 15 | 100 | 0.3 |
| Triadimenol | P | LC | 8.23 | positive | 296.1 ➔ 70.0 | 10 | 298.1 ➔ 70.0 | 10 | 80 | 0.6 |
| Triazophos (hostathion) | P | LC | 8.18 | positive | 314.1 ➔ 162.0 | 19 | 314.1 ➔ 118.9 | 35 | 100 | 0.3 |
| Trichlorfon | P | LC | 4.05 | positive | 256.9 ➔ 109.0 | 12 | 258.9 ➔ 109.0 | 12 | 170 | 1.25 |
| Trifloxystrobin | P | LC | 9.5 | positive | 409.1 ➔ 186.0 | 12 | 409.1 ➔ 145.0 | 52 | 110 | 0.15 |
| Triflumizole | P | LC | 9.55 | positive | 346.1 ➔ 278.0 | 4 | 345.9 ➔ 73.0 | 15 | 80 | 0.15 |
| Triflumuron | P | LC | 9.19 | positive | 359.0 ➔ 156.0 | 8 | 359.0 ➔ 139.0 | 32 | 120 | 0.6 |
| Trifluralin | P | GC | 7.26 | positive | 264.0 ➔ 160.0 | 15 | 306.0 ➔ 264.0 | 5 | 70 | 0.6 |
| Trimethoprim | P | LC | 3.08 | positive | 291.2 ➔ 123.0 | 24 | 291.2 ➔ 230.1 | 20 | 162 | 0.6 |
| Triticonazole | M | LC | 8.4 | positive | 318.1 ➔ 70.1 | 33 | 320.1 ➔ 70.1 | 16 | 110 | 1.25 |
| Tylmicosin | P | LC | 5.52 | positive | 869.6 ➔ 174.1 | 48 | 869.6 ➔ 696.4 | 44 | 294 | 2.5 |
| Tylosin | M | LC | 6.85 | positive | 916.5 ➔ 174.1 | 40 | 916.5 ➔ 772.4 | 28 | 210 | 1.25 |
| Vinclozolin | P | GC | 9.09 | positive | 212.0 ➔ 145.0 | 25 | 212.0 ➔ 109.0 | 50 | 70 | 0.15 |
| Warfarin | AR | LC | 7.86 | negative | 307.1 ➔ 161.1 | 20 | 307.1 ➔ 250.1 | 20 | 140 | 0.3 |
| Zoxamide | P | LC | 9.03 | positive | 336.0 ➔ 187.1 | 25 | 187.1 ➔ 88.9 | 40 | 200 | 0.3 |

CE: Collision Energy

a ﻿P (pesticide); M (medicament); POP (persistent organic pollutant); AR (anticoagulant rodenticide)

b Gas chromatography (GC) or liquid chromatography (LC), both coupled with tandem triple quadrupole mass spectrometry
